# Exploring new animal models of ulcerative colitis: evaluating chemical and patient-derived microbial triggers to advance translational relevance

**DOI:** 10.1186/s42826-026-00283-9

**Published:** 2026-06-08

**Authors:** Maria Rynikova, Sona Gancarcikova, Stanislav Lauko, Dagmar Mudronova, Petra Adamkova, Martin Janicko, Vlasta Demeckova

**Affiliations:** 1 Department of Animal Physiology, Institute of Biology and Ecology, Faculty of Science, Pavol Jozef Safarik University in Kosice, Kosice, Slovakia; 2 Department of Microbiology and Immunology, University of Veterinary Medicine and Pharmacy in Kosice, Kosice, Slovakia; 3 2nd Department of Internal Medicine, Faculty of Medicine, Pavol Jozef Safarik University in Kosice, Kosice, Slovakia

**Keywords:** Microbiome dysbiosis, Animal model, Ulcerative colitis, Pseudo germ-free mice, Faecal microbiota transplantation, Dextran sodium sulphate

## Abstract

**Background:**

Ulcerative colitis (UC) is a chronic inflammatory disease of the colon with multifactorial aetiology involving genetic, immune, environmental, and microbial factors. Alterations in the gut microbiome are a consistent feature of UC, yet their causal contribution to disease onset and progression remains unresolved. Current animal models rely largely on chemical or genetic induction and fail to capture the complexity of host-microbiome interactions characteristic of human disease. To address this limitation and enhance the translational relevance of preclinical research, this study employed patient-derived microbiota to model UC-associated dysbiosis and investigated its effects alone and in combination with chemical induction.

**Results:**

We compared three mouse models using different UC-induction triggers: dextran sulphate sodium (DSS), faecal microbiota transplantation (FMT) from a UC patient, and their combination (COMB). DSS and COMB treatments induced marked clinical symptoms, whereas FMT alone caused only mild changes, likely due to the short exposure period. Immunophenotyping revealed distinct immune profiles across all models, with leukocyte and neutrophil infiltration in the colonic mucosa of all groups, demonstrating that the microbiota alone can elicit localized immune activation. Transcriptomic analysis showed that FMT significantly modulated tight junction and mucin gene expression and induced microbiome shifts resembling those observed in human UC. In contrast, DSS triggered a strong pro-inflammatory transcriptional response and reduced microbial diversity, but with compositional changes mostly opposing those seen in UC patients. The COMB model combined features of both approaches – producing clinical symptoms and inflammatory activation similar to DSS and tight junction dysregulation resembling FMT.

**Conclusions:**

This study investigated novel experimental models of ulcerative colitis by incorporating patient-derived microbiota as an inducing factor. DSS induced strong clinical and inflammatory responses, FMT primarily altered barrier gene expression and microbiome composition, and their combination merged both inflammatory and epithelial characteristics. These microbiota-based models show promise for more accurately reproducing UC pathophysiology and thereby improving translational relevance. Further optimization is needed, including adjustment of exposure duration and sequence of induction, as well as validation for reproducibility.

**Supplementary Information:**

The online version contains supplementary material available at 10.1186/s42826-026-00283-9.

## Background

Inflammatory bowel diseases (IBD) are chronic, multifactorial disorders of the gastrointestinal tract (GIT). While the incidence of IBD has plateaued in Western countries, it continues to rise in East Asia and high-income Asia Pacific regions, with the West maintaining a high prevalence due to improved survival [1]. Ulcerative colitis (UC), one of the two major forms of IBD, is characterized by continuous mucosal inflammation starting in the rectum and confined to the colon, in contrast to the patchy, transmural inflammation seen in Crohn’s disease (CD). UC typically follows a relapsing-remitting course with symptoms such as bloody diarrhoea, abdominal pain, urgency, and tenesmus [2]. The development of UC is complex and not fully elucidated, involving genetic predisposition, environmental factors (e.g. urban lifestyle, diet, pollutants), immune dysregulation, and alterations in the gut microbiome [3]. Given that UC is characterized by chronic immune-mediated inflammation of the colon, a key objective is to identify the triggers of the dysregulated immune response. To investigate the pathogenesis of UC and to test novel therapies, multiple animal models have been developed, each recapitulating different aspects of the disease. Chemically induced models such as dextran sodium sulphate (DSS) damage the epithelium directly, causing barrier breakdown, neutrophil infiltration, ulceration and symptoms such as bloody diarrhoea in a relatively short time frame [4]. Hapten models, including trinitrobenzene sulfonic acid (TNBS) and oxazolone, are used to generate T-cell mediated responses: TNBS tends to produce Th1-polarized inflammation while oxazolone more closely mimics the Th2-skewed cytokine profile seen in UC [5]. Genetically engineered models of spontaneous colitis, such as *Il10*, *Il2*, *Mdr1a* or *Muc2* knockouts, have been invaluable for uncovering gene functions in intestinal immunity and inflammation. While these models reproduce some features of ulcerative colitis, they rely on complete gene loss, which does not reflect the genetic background of human disease. Moreover, the occurrence of spontaneous colitis in over 70 transgenic mouse strains and the identification of more than 160 IBD susceptibility loci in humans highlight the multifactorial nature of inflammatory bowel disease, driven by complex interactions between genetic, microbial, and environmental factors [5–7]. Most of the established UC models lack the complex microbial stimulus that may be a key player in the UC pathogenesis. Several strands of evidence suggest there is a close relationship between the pathogenesis of UC and intestinal microbiome, especially the disruption of its homeostasis. To this date, numerous studies have reported differences between the gut microbiome of healthy individuals and UC patients, including an overall reduction in microbial diversity [8], decreased stability [9] and shifts in key taxa [10]. These changes have been linked to disease severity [11] and treatment response [12]. To investigate host–microbiome interactions in colitis, several infection-based approaches have been developed. These models, such as those using *Salmonella typhimurium*, adherent-invasive *Escherichia coli* or *Helicobacter hepaticus*, can provide insight into host-pathogen interactions and acute intestinal inflammation. However, *S. typhimurium* inoculation often leads to systemic infection [13] and *H. hepaticus* is efficient in immunologically manipulated mice [14]. While these models reproduce certain features of UC, they fail to recapitulate the complex microbial alterations observed in human disease.

There is a clear need for relevant preclinical models that more faithfully reproduce not only the immunological but also the microbial features of UC, thereby increasing their translational relevance for therapy development. Therefore, the aim of this study was to investigate faecal microbiota transplantation (FMT) from a UC patient as a microbial trigger of colitis, and to compare it with the widely used DSS model as well as their combination, in order to establish a more translationally relevant model that better captures the complexity of UC.

## Methods

### Animal husbandry and experimental conditions

The presented study was carried out on 81 specific pathogen-free (SPF) female BALB/c mice, 6 weeks old (19.9 ± 1.3 g) obtained from Velaz s.r.o. (Prague, Czech Republic). Upon arrival, the mice were housed in gnotobiotic conditions (Laboratory of Gnotobiology of the Department of Microbiology and Immunology, UVMP Kosice, Slovakia, SK U 16016). The animals were provided *ad libitum* access to irradiated feed and sterile water, with regular bedding changes. All equipment was sterilized by autoclaving or gamma-irradiation and further disinfected with 2% peracetic acid before use. Detailed housing and care procedures are provided in the Additional file 1.

### Antibiotic decontamination

To generate a pseudo germ-free mouse model with a substantially reduced and controlled microbiota, while maintaining the overall health status of the animals, all experimental mice underwent a 5-day antibiotic treatment followed by a 10-day convalescence period. This approach was based on the protocol described by Popper et al. (2016) [15] and involved administering amoxicillin and clavulanic acid (Amoksiklav; Sandoz Pharmaceuticals, Ljubljana, Slovenia) and ciprofloxacin (Ciprinol con infusion; Krka d.d., Novo Mesto, Slovenia) every 12 hours. The effectiveness of this model in achieving microbiota depletion and its reliability for downstream experimental applications has been confirmed in several published studies [16, 17].

### Donor selection and FMT preparation

The donor evaluation included anamnesis, clinical history, and laboratory testing. The selected donor was a 20-year-old female diagnosed with acute UC (Mayo score = 2) with no history of prior antibiotic, corticosteroid, or biological therapy. The stool sample for FMT preparation was processed within 6 months of the screening and no more than 6 hours post-collection. The FMT preparation protocol followed the method outlined by Lauko et al. [16]. A detailed description of the donor selection and FMT preparation process is provided in the Additional file 1.

### Colitis induction

To achieve pseudo germ-free status, all intact animals (INT) were subjected to antibiotic treatment. After the decontamination, at the beginning of the experiment, a subset of these animals was sacrificed to serve as the control group (C0). The remaining animals were divided into three experimental groups based on the method of UC induction. The DSS group (*n* = 22) received 5% dextran sulphate sodium (DSS, molecular weight 40 kDa, TdB Consultancy AB, Uppsala, Sweden) in drinking water for 5 days. The FMT group (*n* = 30) underwent UC induction through oral administration of UC-derived FMT every 24 hours for 5 days. The COMB group (*n* = 29) was subjected to a combination of the two methods, receiving DSS in drinking water and FMT orally, both administered over 5 days. At the end of the experiment, all animals were euthanized by cervical dislocation. No anaesthesia was used during this procedure or any other procedure performed during the experiment.

### Evaluation of clinical colitis

The severity of the disease and clinical symptoms were assessed using an updated and revised version of Disease Activity Index (DAI) [15]. This index combines scores for rectal bleeding (0–6), stool consistency (0–6), and weight loss (0–4) (Table 1). Rectal bleeding was assessed by an anal smear with a moistened cotton swab and scored according to the blood coverage of the swab.

**Table 1:** Disease activity index (DAI). DAI was calculated as the sum of rectal bleeding, stool consistency, and weight loss scores

Score	Rectal Bleeding	Stool Consistency	Weight Loss	Maximum Score
0	No bleeding	Formed pellets	<5%	16
1			5–10%	
2	Spotting	Mild soft	11–15%	
3			16–20%	
4	Slight bleeding	Very soft	>20%	
5			-	
6	Gross bleeding	Watery stool	-	

### Immunophenotyping of secondary lymphoid organs

The splenocytes were isolated by perforating the spleen, flushing with HBSS, and centrifuging the resulting suspension. For isolation of cells from Peyer’s patches, tissue was digested with Collagenase from *Clostridium histolyticum* in RPMI 1640 medium (both from Sigma-Aldrich, St. Louis, MO, USA), filtered, and processed similarly to the splenocytes. Colon tissue was processed into a single-cell suspension by digestion with Collagenases (II and IV) and Hyaluronidase IV (all from Worthington Biochemical Corporation, Lakewood, NJ, USA), followed by filtration and centrifugation. Cell recovery was determined using the MUSE® cell analyser (Cytek Biosciences, Fremont, CA, USA) with the Muse® Count & Viability Kit (Luminex, Austin, TX, USA). A detailed description of the methods used to obtain the single-cell suspensions is provided in the Additional file 1. Single cell suspensions from secondary lymphoid organs were stained with fluorescent-labelled monoclonal antibodies (eBioscience, San Diego, CA, USA; Additional files 2 and 3). The stained samples were analysed using the BD FACSCanto™ flow cytometer (Becton Dickinson Biosciences, Franklin Lakes, NJ, USA) and BD FACS Diva™ (Becton Dickinson Biosciences, Franklin Lakes, NJ, USA) and FlowLogic software (Version 7.3.; Inivai Technologies, Mentone, Victoria, Australia). In each of the analysed secondary lymphoid organs we assessed populations of leukocytes, T lymphocytes, and B lymphocytes. In the colon mucosa specifically we also evaluated macrophages, neutrophils, natural killer (NK) cells, and NK T cells (NKT). The gating strategy followed the standardized and validated protocol described by Bayne and Vonderheide [18], and the analysed cell populations and the specific markers used to define them are detailed in Table 2.

**Table 2:** Markers of immune cell population in secondary lymphoid organs. Populations listed without indentation represent parent populations, while indented entries indicate their respective subpopulations

Cell population	Markers (colon mucosa)	Markers (spleen and PP)
Leukocytes	CD45+	CD45+
Myeloid cells	CD11b+	
Macrophages	F4/80+Ly-6G-	
Neutrophils	F4/80-Ly-6G+	
T-lymphocytes	CD3+CD19-	CD3+B-220-
Helper T cells (Th)	CD4+CD8a-	CD4+CD8a-
Cytotoxic T cells (Tc)	CD4–CD8a+	CD4–CD8a+
B-lymphocytes	CD3–CD19+	CD3–B220+
NK cells	CD8a-CD49b+	
NKT cells	CD8a+CD49+	

### DNA and RNA extraction

Faecal samples were collected throughout the experiment and stored at −70 °C until further processing. Genomic DNA was isolated using the ZR Fecal DNA MiniPrep™ kit (Zymo Research, Irvine, CA, USA) following the manufacturer’s protocol. Colon tissue samples (∼ 2 cm) were excised post-mortem, snap-frozen in liquid nitrogen and stored at −70 °C. For RNA extraction, frozen tissues were transferred directly into gentleMACS™ M Tubes (Miltenyi Biotec, Bergisch Gladbach, Germany) containing TRI Reagent® (Molecular Research Center, Cincinnati, OH, USA) without allowing them to thaw. The tissues were homogenized using the gentleMACS™ Dissociator (Miltenyi Biotec, Bergisch Gladbach, Germany). Subsequently, mucosal RNA was isolated from the homogenized tissue using the Monarch® Total RNA Miniprep Kit (New England Biolabs, Ipswich, MA, USA) according to the manufacturer’s protocol.

### Evaluation of microbial composition (16S sequencing) and transcriptomic analysis of colon mucosa (RNA-Seq)

The concentration and purity of the isolated DNA and RNA samples were first assessed in-house using a spectrophotometer (NanoDrop™ One/OneC, Thermo Fisher Scientific, Waltham, MA, USA). The samples were then shipped on dry ice to Novogene Europe (Cambridge, UK) for library preparation, sequencing, data processing, and statistical analysis (Additional file 1).

We focused our transcriptome analysis on genes associated with tight junction proteins and mucins, given the proposed central role of the intestinal epithelial barrier (IEB) disruption in UC pathogenesis. Additionally, genes for cytokines and chemokines, previously linked to inflammation in UC, were also analysed. A panel of 114 genes (Additional file 4) was curated from the published literature and examined for differential expression.

### Statistical analysis

Unless stated otherwise, all statistical analyses were performed using GraphPad Prism 9 (GraphPad Software, San Diego, CA, USA). The normality of data distributions was assessed using the Shapiro-Wilk test, and the equality of variances was evaluated using the Brown-Forsythe test. For normally distributed data with equal variances, an unpaired two-tailed Student’s t-test or one-way ANOVA with Tukey’s post hoc test were applied for two-group and multiple-group comparisons, respectively. In cases where the normality criterion was met but variances were unequal, Welch’s t-test or Welch’s ANOVA followed by Dunnett’s T3 multiple comparison test were used. Non-parametric data were analysed using the Mann-Whitney U test for two-group comparisons or the Kruskal-Wallis test with Dunn’s post hoc test for multiple groups. The Disease Activity Index, as ordinal repeated measures data, was analysed using the Friedman test followed by Dunn’s multiple comparison test. A Bonferroni correction for multiple testing was applied to all two-group tests where necessary. On selected immune cell population data, a Spearman’s rank correlation was performed post hoc.

## Results

### DSS alone induces the highest Disease Activity Index (DAI) among UC induction methods

**Fig. 1:**
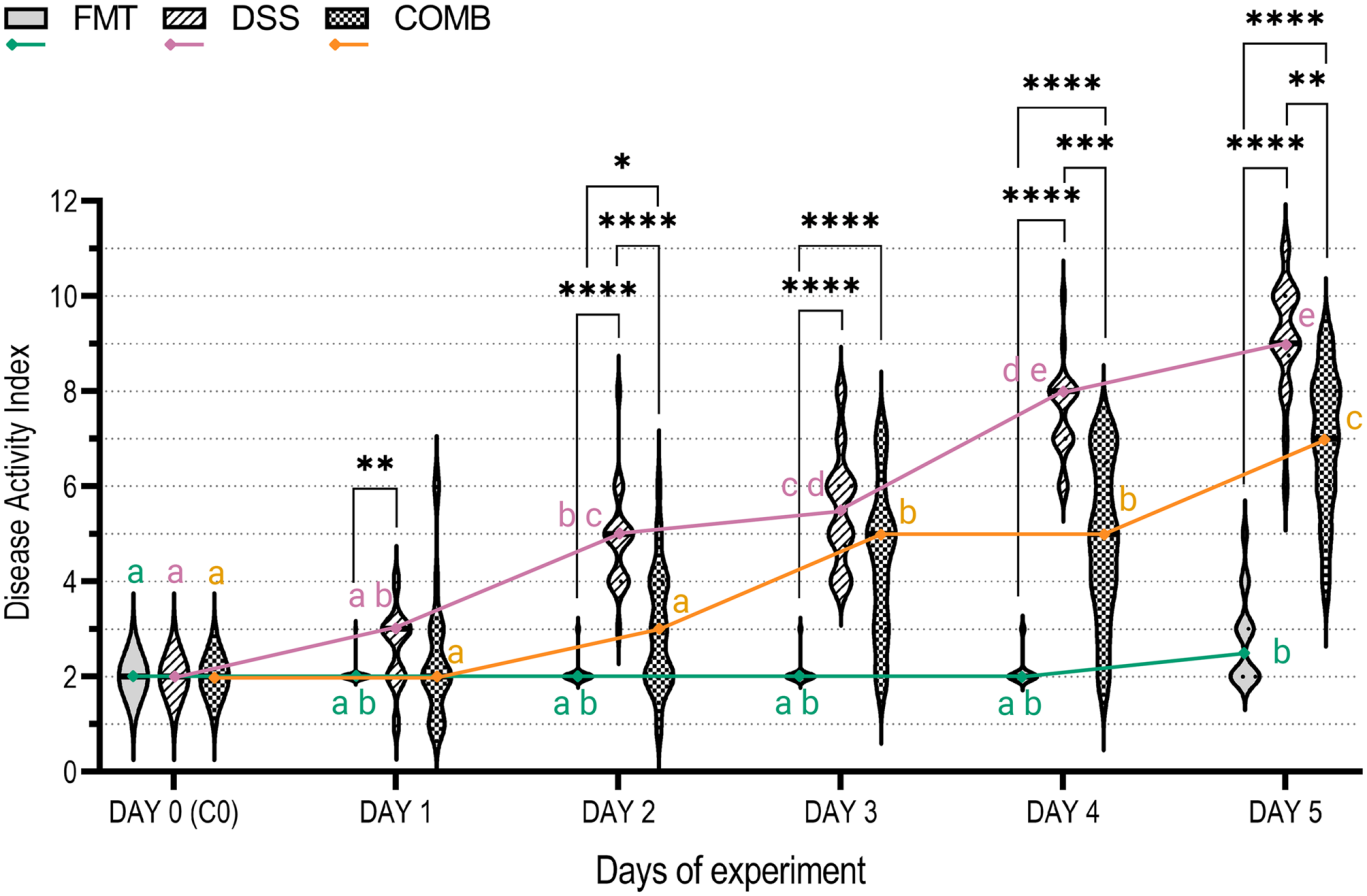
Timeline of disease activity index across the experiment. Intergroup pairwise comparisons for each experimental day were performed using the Kruskal–Wallis test followed by Dunn’s multiple comparisons test and are depicted with asterisks. Statistical significance levels are denoted as follows: *p < 0.05, **p < 0.01, ***p < 0.001, and ****p < 0.0001. Intragroup pairwise comparisons between experimental days were conducted using Friedman’s test with Dunn’s multiple comparisons test and are indicated with color-coded letter annotations

A significant increase in the DAI score was observed across all groups during the induction period (Fig. 1). The FMT group developed only mild disease, with a slight rise in DAI detectable at the end of the induction period (final DAI ≈2.5). In contrast, DSS treatment caused an early and steadily progressive increase in DAI from the first day of induction, resulting in the most severe clinical phenotype (final DAI ≈9). The COMB group followed an intermediate course: animals developed more pronounced clinical symptoms than in the FMT group, but the increase in DAI remained consistently lower than in the DSS group (final DAI ≈7). At the end of the induction period, DAI values were highest in DSS-treated mice, followed by COMB and then FMT, confirming that the three protocols model distinct grades of clinical disease severity.

### UC induction methods alter immune cell populations in secondary lymphoid organs in a distinct manner

**Fig. 2:**
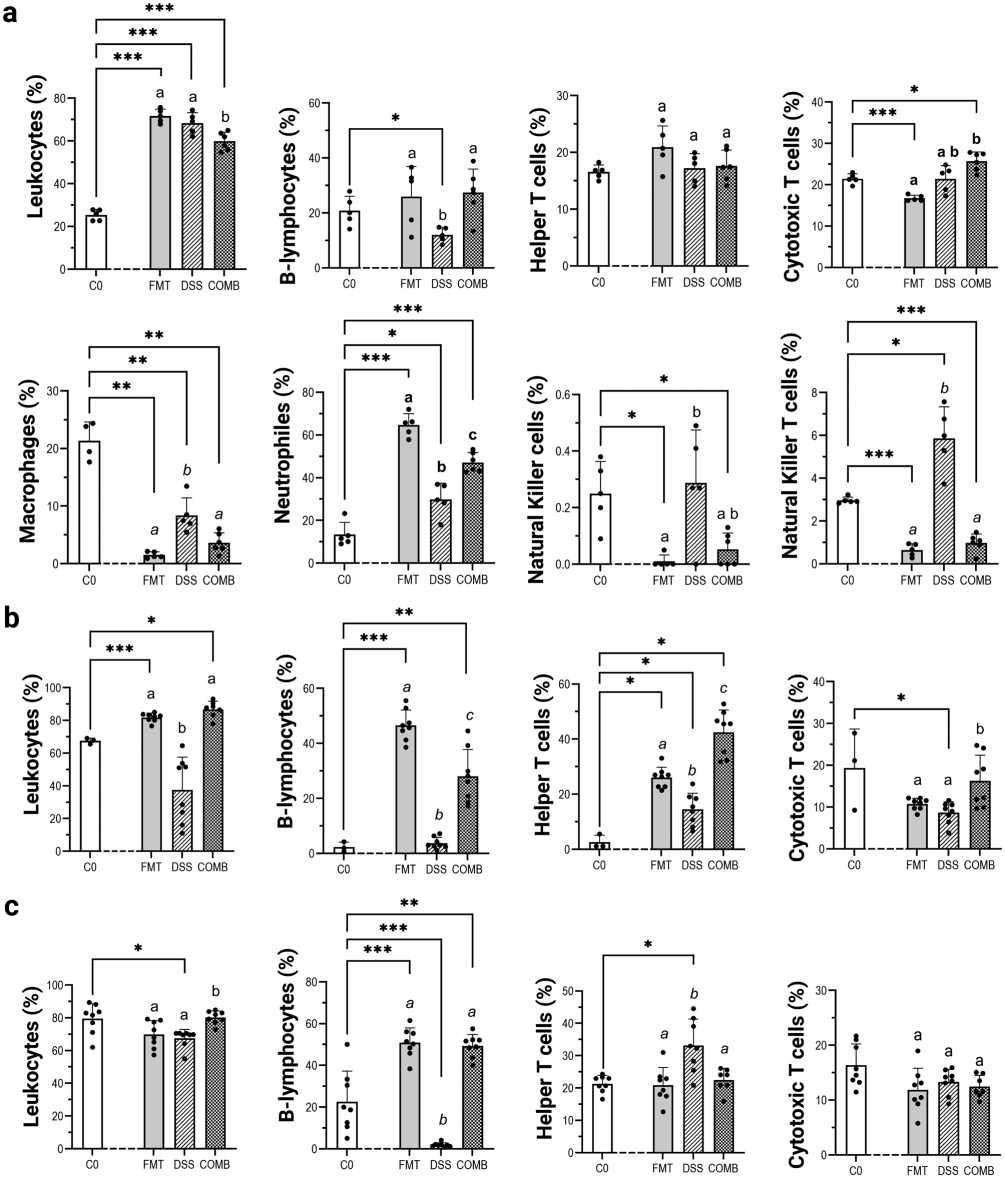
Immune cell populations of secondary lymphoid organs: (**a**) Colon mucosa. (**b**) Peyer’s patches. (**c**) Spleen. Data are presented as mean + SD and individual data points. Comparisons between control (day 0; C0) and post-UC induction were performed using t-tests with Bonferroni correction applied (α’ = α/3), and statistical significance is indicated by asterisks. Intergroup pairwise comparisons between UC induction methods (FMT, DSS, and COMB groups) were analysed using parametric or nonparametric tests based on data distribution and variance homogeneity, followed by appropriate multiple comparison tests (see methods section). These comparisons are represented by letters; identical letters indicate no statistical significance, while different letters denote significant differences. Levels of significance are denoted as follows: for t-tests (asterisks), *p < 0.0167, **p < 0.0033, ***p < 0.00033; for pairwise comparisons (letters): normal letters (*x*, *y*) for *p* < 0.05, italicized letters (*x*, *y*) for *p* < 0.01, and bold letters (*x*, *y*) for *p* < 0.001

In the colon mucosa (Fig. 2a), leukocyte and neutrophil populations increased across all experimental groups compared to the control, with the most pronounced neutrophil expansion in the FMT group and the least in the DSS group. In contrast, macrophage percentages significantly decreased in all experimental groups. The percentage of NKT cells was also significantly altered but followed different trends: it decreased in the FMT and COMB groups while increasing in the DSS group. No significant differences were observed in helper T-cell (CD4+CD8-) percentages. Additionally, the DSS group exhibited a reduction in B-lymphocytes, while in both the FMT and COMB groups a significant reduction in NK cells and cytotoxic T cells (CD4–CD8+) was observed. Exploratory Spearman’s correlation between NK cells and neutrophils was also conducted post hoc. Results showed there was a strong negative correlation between the percentages of NK cells and neutrophils, r_s_(28) = −0.79, *p* = 0.0009. In Peyer’s patches (Fig. 2b), a possible synergistic effect of DSS and UC FMT was observed in the helper T cell population, which increased significantly in all experimental groups compared to controls, with the highest increase observed in the COMB group. Following UC induction, the DSS group displayed significant changes in T-cell populations, including a decrease of cytotoxic T cells while helper T-cell increased. In the FMT and COMB groups, leukocytes, B-cell and helper T-cell populations increased compared to controls. Comparisons among induction methods revealed significant differences in B-cell, T-cell, and helper T-cell populations across all groups. In the spleen (Fig. 2c), DSS administration caused the most pronounced deviations from the control group, including a decrease in leukocyte and B-lymphocyte percentages, along with an increase in total T cells and helper T cells. In contrast, FMT administration with or without simultaneous DSS administration primarily led to an increase in B-lymphocytes without significant alterations in other cell populations.

### DSS alone induces subtler shifts to transcriptome of colon mucosa cells compared to FMT

**Fig. 3:**
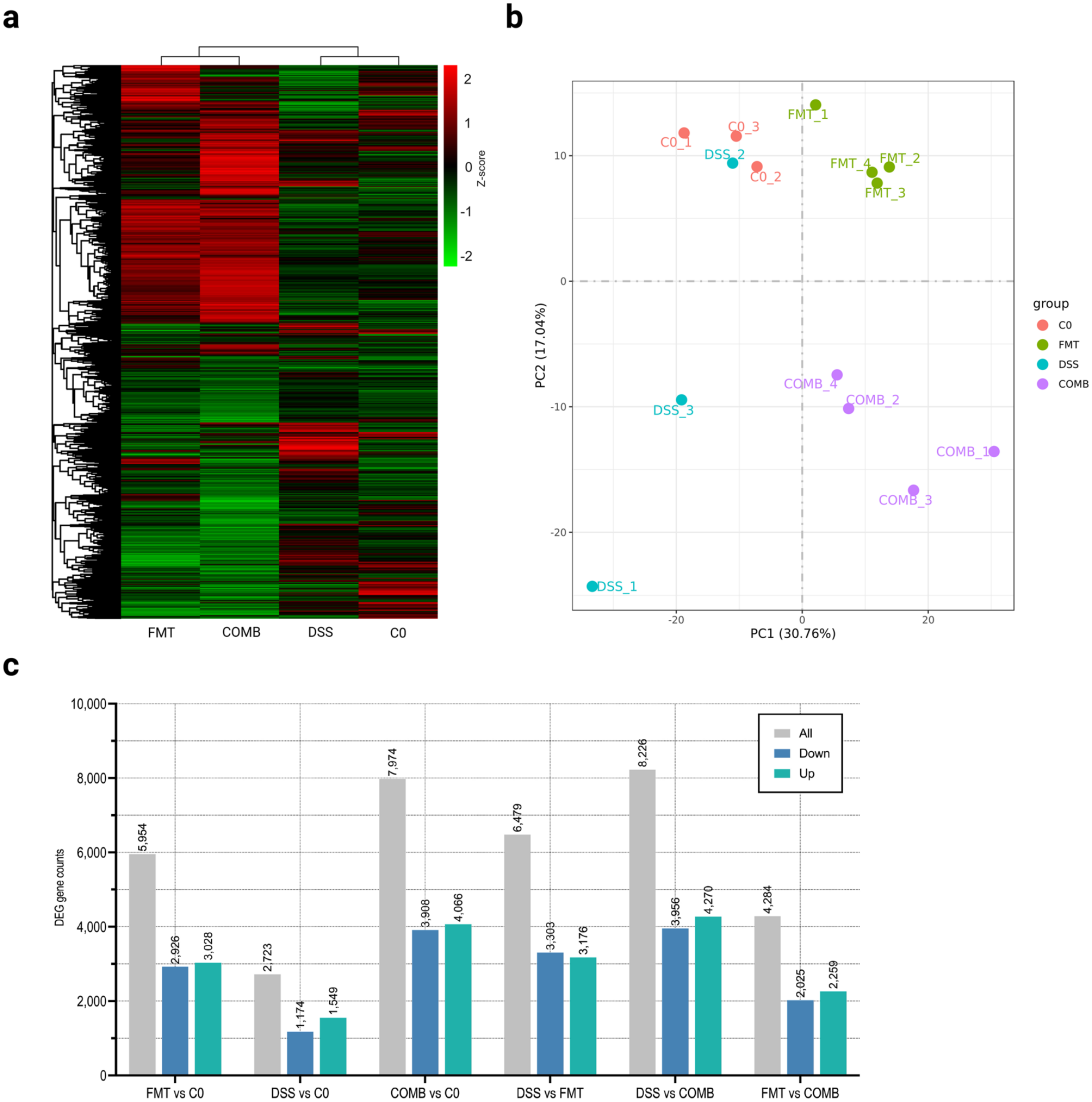
Overview of transcriptomic variability and sample grouping performed as part of the transcriptomic analysis of colon tissue using RNA-seq. (**a**) Hierarchical cluster analysis (HCA). Hierarchical clustering was performed on the log_2_(FPKM+1) values of gene expression. The data were normalized by calculating the Z-score for each row. The heatmap colours represent the normalized expression values, reflecting relative differences within each row. As a result, the colours can only be compared horizontally (within a row) and not vertically (between rows). (**b**) Principal component analysis (PCA). Analysis was performed on the gene expression values (FPKM). (**c**) Statistics of differentially expressed genes between groups

Hierarchical cluster analysis (HCA) (Fig. 3a) revealed that out of all experimental groups, the DSS group was most similar to the control group, with these two groups clustering together and the FMT and COMB groups were clustering separately. Principal Component Analysis (PCA) (Fig. 3b) revealed that samples within the C0 and FMT groups respectively clustered most tightly, indicating low intra-group variability. In contrast, samples from the DSS group exhibited the greatest dispersion and partially overlapped with the C0 group, suggesting higher variability. Differential gene expression analysis supported these findings (Fig. 3c) with DSS and C0 sharing the lowest number (2,723) of differentially expressed genes (DEGs). Among the induction methods, COMB and FMT were the most similar, with 4,284 DEGs, whereas COMB and DSS exhibited the greatest differences, with 8,226 DEGs.

### FMT downregulates key genes associated with tight junctions and mucins while DSS upregulates inflammation-related genes

**Fig. 4:**
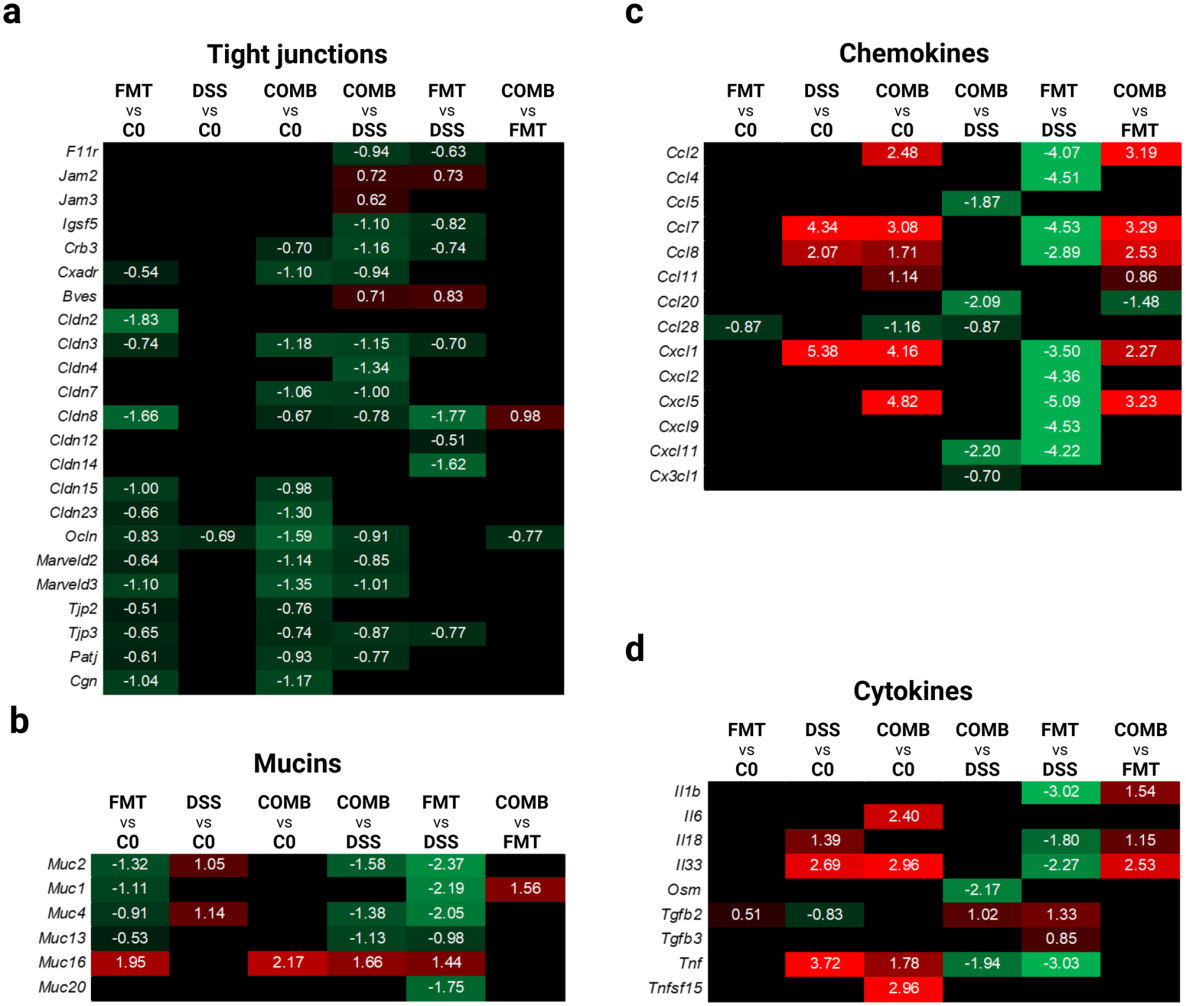
Differential expression of selected gene sets identified as part of the transcriptomic analysis of colon tissue using RNA-seq. (**a**) Tight junctions. (**b**) Mucins. (**c**) Chemokines. (**d**) Cytokines. Genes with a | log_2_ Fold change (FC) |≥0.5 and padj < 0.05 were identified as differentially expressed. The heatmap displays expression levels, with green indicating downregulation (negative log_2_ FC values) and red indicating upregulation (positive log_2_ FC values). Exact log_2_ FC values are shown for all differentially expressed genes

Upon examining the panel of 114 genes for tight junctions, mucins, cytokines and chemokines, 52 were differentially expressed in at least one experimental group (Fig. 4). Administration of DSS alone had the smallest impact on tight junction protein expression (Fig. 4a), with only one gene (*Ocln*) downregulated compared to the control group. In contrast, FMT administration alone or in combination with DSS (COMB group) resulted in downregulation of over half the genes in the tight junction panel and numerous significant differences emerged between the DSS group and the FMT/COMB groups. The impact of FMT on tight junction gene expression was further corroborated by KEGG enrichment analysis. The Tight Junction pathway (mmu04530) was significantly downregulated in both the FMT and COMB groups but not in the DSS group (Table 3, Additional files 5 and 6).

**Table 3:** KEGG enrichment analysis: summary of differential gene expression in the tight junction pathway (mmu04530). DEGs – differentially expressed genes. padj – *p* value adjusted for multiple testing using the Benjamini–Hochberg false discovery rate (FDR) method

Groups	DEGs in the pathway	Gene expression	p value	padj
FMT vs C0	48	Downregulation	0.002567	0.038379
COMB vs C0	58	Downregulation	0.002570	0.030179
DSS vs C0	29	Upregulation	0.473748	0.743559

In the mucin panel (Fig. 4b), contrasting effects of FMT and DSS administration were observed. Most genes were downregulated in the FMT group compared to the control, whereas *Muc2* and *Muc4* were upregulated in the DSS group. In the COMB group, no genes were differentially expressed except for *Muc16*, which was significantly upregulated. These findings were further supported by KEGG enrichment analysis (Table 4). The Mucin Type O-glycan Biosynthesis pathway (mmu00512) was significantly down-regulated in the FMT group, while it was upregulated in the DSS group. No significant changes were observed in the COMB group. Similar to the findings in the tight junction panel, notable differences emerged between the DSS group and the FMT/COMB groups.

**Table 4:** KEGG enrichment analysis: summary of differential gene expression in the mucin type O-glycan biosynthesis pathway (mmu00512). DEGs – differentially expressed genes. padj – *p* value adjusted for multiple testing using the Benjamini–Hochberg false discovery rate (FDR) method

Groups	DEGs in the pathway	Gene expression	p value	padj
FMT vs C0	12	Downregulation	0.000688	0.014397
COMB vs C0	9	Downregulation	0.080581	0.311515
DSS vs C0	9	Upregulation	0.000185	0.005169

In the chemokine (Fig. 4c) and cytokine (Fig. 4d) panels, similar trends were observed. The FMT group exhibited the fewest DEGs after induction, with only *Ccl28* and *Tgfb2* differentially expressed in the chemokine and cytokine panels, respectively. After DSS administration alone, several genes were upregulated, and one gene, *Tgfb2*, was downregulated. In contrast, the COMB group showed the highest number of DEGs, with all being upregulated except for *Ccl28*, which was downregulated. KEGG Enrichment analysis revealed significant upregulation of several cytokine and chemokine-related pathways in the DSS group compared to the control (Table 5).

**Table 5:** KEGG enrichment analysis: upregulation of selected pathways in the DSS group (versus C0). DEGs – differentially expressed genes. padj – *p* value adjusted for multiple testing using the Benjamini–Hochberg false discovery rate (FDR) method

KEGG ID	Pathway name	DEGs in the pathway	p value	padj
mmu04060	Cytokine-cytokine receptor interaction	48	7.9E-09	1.21E-06
mmu04668	TNF signaling pathway	26	2.66E-06	2.15E-04
mmu04657	IL-17 signaling pathway	20	6.56E-05	2.51E-03
mmu04062	Chemokine signaling pathway	30	4.2E-04	8.64E-03

In contrast, the COMB group showed significant upregulation of only the Cytokine-cytokine receptor interaction pathway (mmu04060) compared to the control, while no cytokine or chemokine-related pathways were significantly upregulated in the FMT group.

**Fig. 5:**
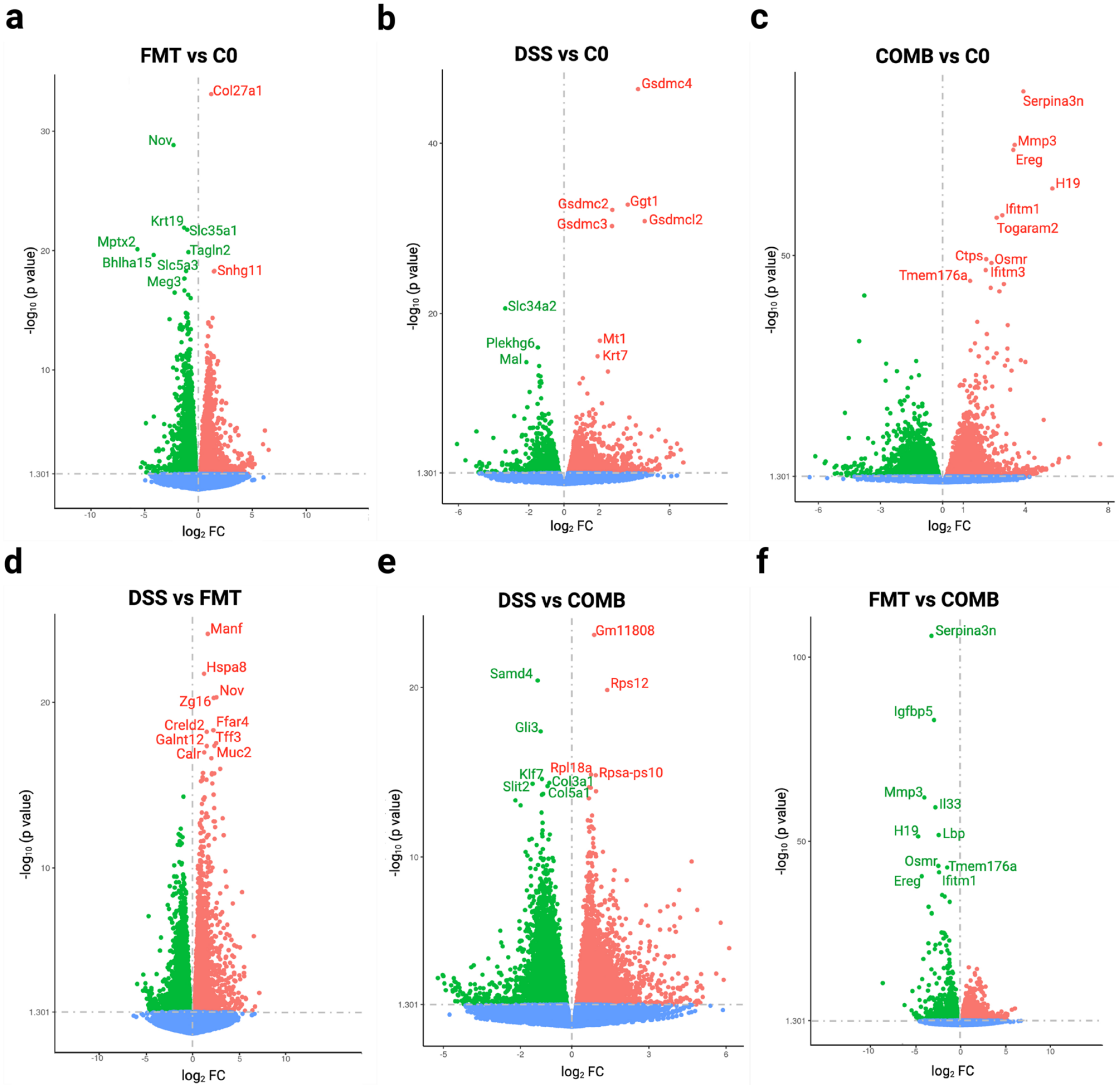
Volcano plots of differentially expressed genes identified as part of the transcriptomic analysis of colon tissue using RNA-seq. (**a**) FMT group vs control group. (**b**) DSS group vs control group. (**c**) COMB group vs control group. (**d**) DSS group vs FMT group. (**e**) DSS group vs COMB group. (**f**) FMT group vs COMB group. Up-regulated genes are depicted in pink, down-regulated genes in green, and non-differentially expressed genes in blue. Each dot represents an individual gene. The top ten most significant genes in each volcano plot, determined by the lowest *p*-values, are labelled with their respective gene names

The analysis of top 10 most differentially expressed genes, further revealed distinct transcriptional patterns among the experimental groups. In the FMT group (Fig. 5a), *Col27a1* and *Snhg11* were upregulated, while several genes including *Nov*, *Krt19*, *Slc35a1*, *Mptx2*, *Tagln2*, *Bhlha15*, *Slc5a3*, and *Meg3* were downregulated compared to controls. The DSS group (Fig. 5b) showed marked upregulation of pyroptosis- and stress-related genes such as *Gsdmc4*, *Ggt1*, *Gsdmc2*, *Gsdmcl2*, *Gsdmc3*, *Mt1*, and *Krt7*, alongside downregulation of *Slc34a2*, *Plekhg6*, and *Mal* compared to the control group. In the COMB model (Fig. 5c), when compared to C0, all top 10 DEGs were upregulated (*Serpina3n*, *Mmp3*, *Ereg*, *H19*, *Ifitm1*, *Togaram2, Ctps, Osmr*, *Ifitm3* and *Tmem176a*).

When comparing the models directly, DSS vs FMT revealed higher expression of *Manf*, *Hspa8*, *Nov*, *Zg16*, *Ffar4*, *Creld2*, *Tff3*, *Galnt12*, *Muc2*, and *Calr* in DSS, while DSS vs COMB showed upregulation of *Gm11808*, *Rps12*, *Rpl18a*, and *Rpsa-ps10*, and downregulation of *Samd4*, *Gli3*, *Klf7*, *Col3a1*, *Slit2*, and *Col5a1* in the DSS group. Lastly, FMT vs COMB comparison revealed consistent downregulation of the top 10 DEGs in the FMT group, including *Serpina3n*, *Igfbp5*, *Mmp3*, *Il33*, *Lbp*, *H19*, *Osmr*, *Tmem176a*, *Ifitm1*, and *Ereg*.

### DSS reduces microbiome diversity, even in combination with FMT

**Fig. 6:**
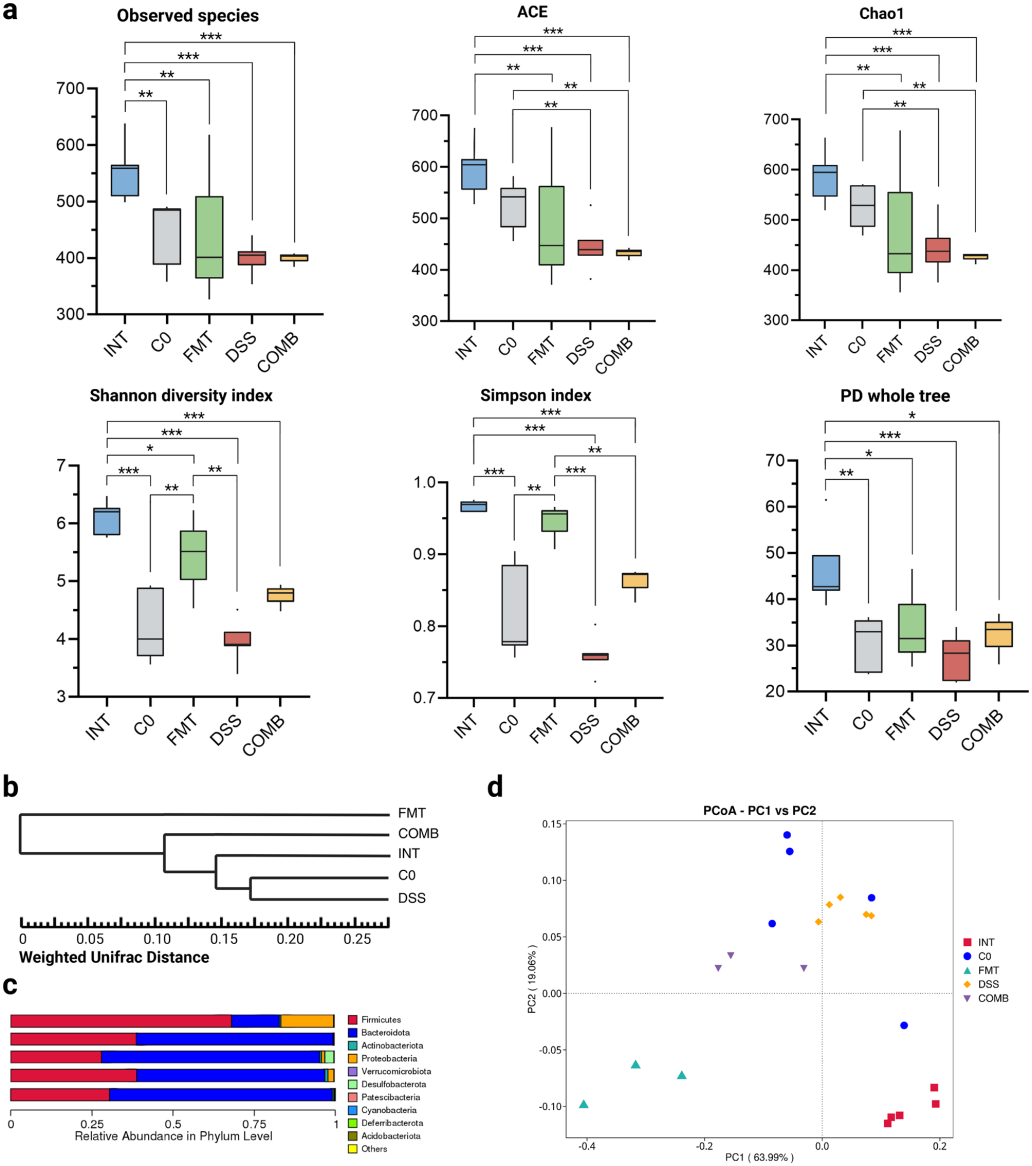
Alpha and beta diversity analyses of faecal microbiota based on 16S rRNA sequencing. (**a**) Alpha diversity indices. Data are presented as median with range. Comparisons between groups were performed using Wilcoxon test and statistical significance is indicated by asterisks. Statistical significance is indicated by asterisks. *p < 0.05, **p < 0.01, ***p < 0.001. (**b**, **c**) UPGMA (Unweighted pair group method with arithmetic mean): (**b**) Dendrogram depicting hierarchical clustering of groups. (**c**) Bar plots displaying the relative abundance of the bacterial phyla used for clustering. d PCoA (Principal coordinates analysis) plot based on weighted UniFrac distances

To comprehensively assess changes in alpha diversity (Fig. 6a), experimental groups were compared not only to the control group (C0) but also to intact animals prior to antibiotic treatment (INT). Following antibiotic treatment, the number of observed species significantly decreased and was not restored by any of the UC induction methods, indicating reduced microbial diversity. No significant changes in the number of observed species were observed after ulcerative colitis induction compared to C0 while species richness, as measured by ACE (Abundance-based coverage estimator) and Chao1, significantly decreased in the DSS and COMB groups compared to both INT and C0. However, in the FMT group, species richness was significantly reduced only when compared to INT. Diversity metrics such as Shannon, Simpson, and PD whole tree also showed significant reductions in the DSS and COMB groups compared to INT but not relative to C0. In contrast, Shannon and Simpson indices significantly increased in the FMT group compared to C0, suggesting enhanced diversity. While the Simpson index in the FMT group remained unchanged relative to INT, both the Shannon index and PD whole tree were significantly lower compared to INT. Between the experimental groups, the only observed differences were in the Simpson index, which was significantly lower in the DSS and COMB groups compared to FMT, and the Shannon index, which was significantly lower in DSS compared to FMT.

### Beta diversity analysis reveals greater similarity between COMB and DSS groups compared to FMT

UPGMA (Unweighted Pair Group Method with Arithmetic Mean, Fig. 6b, c) and PCoA (Principal Coordinates Analysis; Fig. 6d) both indicated that the DSS and C0 groups had the most similar microbial community compositions, as they clustered closely in UPGMA and exhibited overlapping samples in PCoA. These findings were supported by Adonis (PERMANOVA) and MRPP (Multi-Response Permutation Procedure) (Table 6), though the COMB group showed similarly low F and A statistics, indicating moderate differentiation.

**Table 6:** Community differences analyses: Adonis (PERMANOVA) and MRPP (Multi-response permutation procedure)

	**INT** vs				**C0** vs			FMT vs **DSS**	FMT vs **COMB**	COMB vs **DSS**
	C0	FMT	DSS	COMB	FMT	DSS	COMB			
**Adonis**										
F	15.78	19.64	43.26	24.80	10.28	6.18	4.34	33.74	18.88	9.08
R^2^	0.66	0.77	0.84	0.81	0.63	0.43	0.42	0.85	0.83	0.60
*p* value	0.001**	0.017*	0.001**	0.015*	0.001**	0.001**	0.039*	0.001**	**0.100**	0.023*
**MRPP**										
A	0.38	0.43	0.56	0.50	0.34	0.25	0.25	0.54	0.52	0.31
*p* value	0.005**	0.020*	0.014*	0.017*	0.020*	0.011*	0.025*	0.023*	**0.100**	0.015*

F-statistic, R^2^, and *p*-values from Adonis; A-statistic and *p*-values from MRPP are presented across different group comparisons. Statistical significance is indicated by asterisks. *p < 0.05, **p < 0.01, ***p < 0.001

In contrast, the FMT group demonstrated the most distinct microbial composition. It was clustered farthest from other groups in PCoA and only joined the main UPGMA cluster at the final stage. Based on the Adonis and MRPP results, the FMT group exhibited more pronounced differences when compared to DSS and COMB groups, as indicated by relatively high F and A statistics, but only moderate differentiation when compared to the C0.

### Distinct bacterial phyla and families shape the microbiome following different UC induction methods

**Fig. 7:**
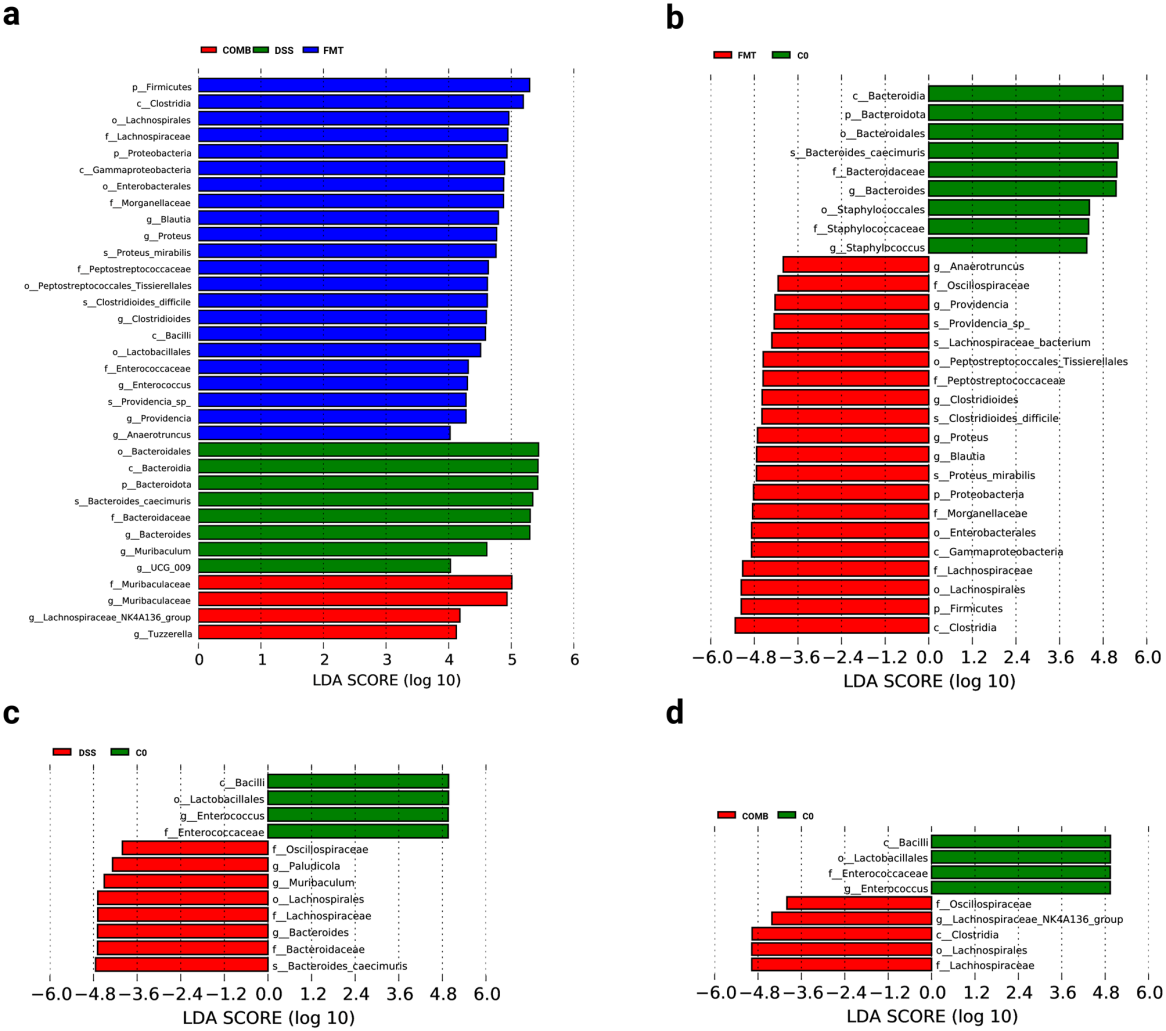
LEfSe analysis of faecal microbiota based on 16S rRNA sequencing. (**a**) LEfSe analysis comparing microbial biomarkers among experimental groups (FMT, DSS, and COMB). (**b**–**d**) LEfSe analysis of each experimental group compared to the control group: (**b**) FMT group vs control group. (**c**) DSS group vs control group. (**d**) COMB group vs control group

**Fig. 8:**
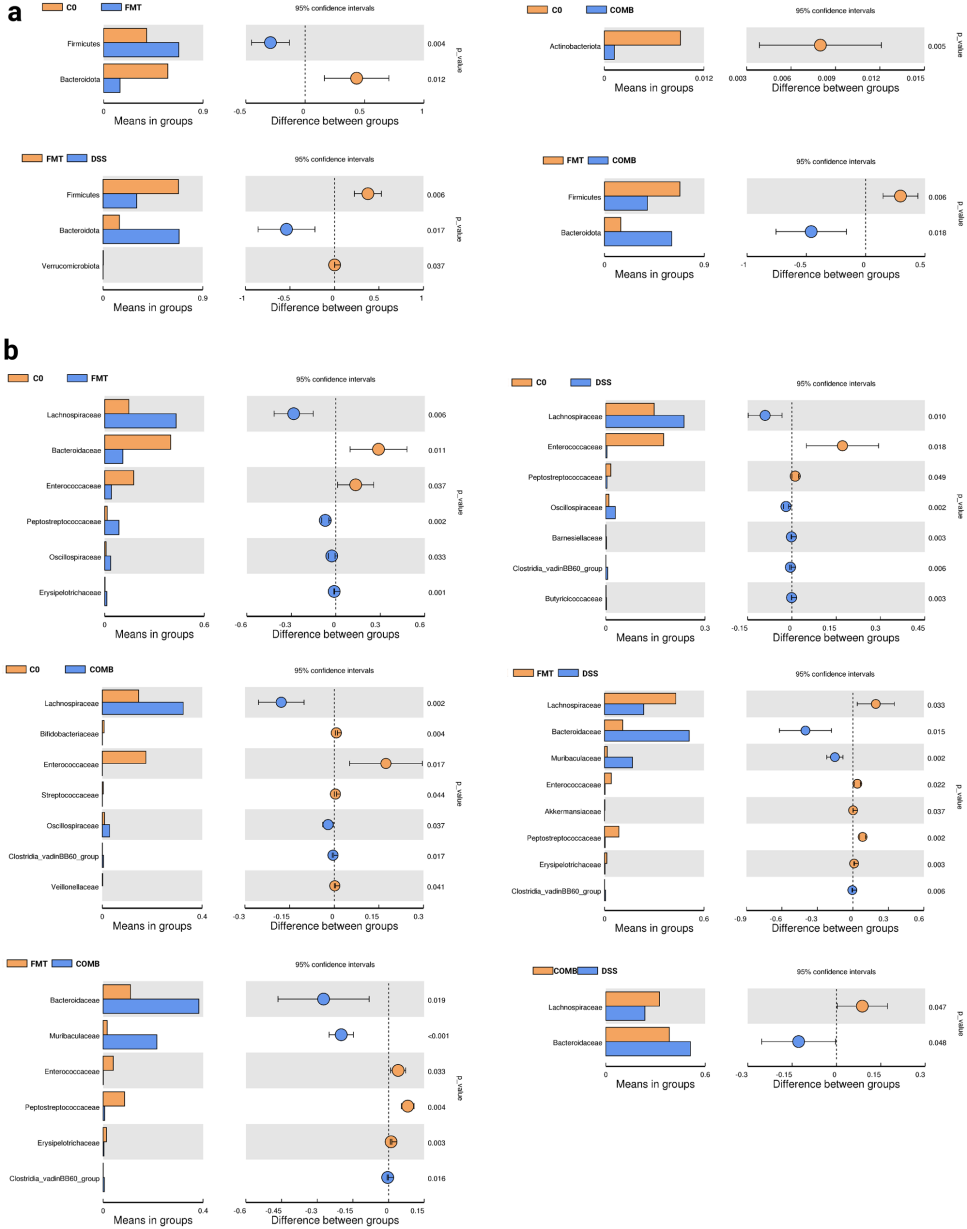
Bar plots of Student’s t-tests comparing bacterial populations of faecal microbiota based on 16S rRNA sequencing. (**a**) Significant differences at the phylum level. (**b**) Significant differences at the family level. Bar plots of mean relative abundance are shown on left and 95% confidence intervals for group differences shown on right

Upon comparing the methods of UC induction, LEfSe (Linear discriminant analysis Effect Size) analysis identified distinct microbial taxa as biomarkers for each experimental group, with significant differences observed across multiple taxonomic levels (Fig. 7a). The FMT group exhibited the highest number of biomarkers (22), while the COMB group had the fewest (4). For the FMT group, biomarkers encompassed entire phyla (Firmicutes and Proteobacteria), alongside several subordinate taxa: classes (Clostridia, Gammaproteobacteria, Bacilli), orders (Lachnospirales, Enterobacterales, Peptostreptococcales-Tissierellales, Lactobacillales), families (Lachnospiraceae, Morganellaceae, Peptostreptococcaceae, Enterococcaceae), and genera (*Blautia, Proteus, Clostridioides, Enterococcus, Providencia, Anaerotruncus*). At the species level, *Proteus mirabilis* and *Clostridioides difficile* were unique biomarkers of this group. This trend was also evident when comparing the FMT group to the control (C0) (Fig. 7b), where Firmicutes and Proteobacteria phyla, along with their subordinate taxa, were biomarkers of the FMT group. Similarly to the control group, the DSS group was characterized by biomarkers predominantly from the Bacteroidota phylum (Bacteroidia, Bacteroidales, Bacteroidaceae, Muribaculaceae, *Bacteroides, Muribaculum, UCG-009*, *Bacteroides caecimuris*). When comparing the C0 and DSS groups (Fig. 7c), the control group was characterized by biomarkers from the Firmicutes phylum, while the *DSS* group exhibited a mix of subordinate taxa from both Firmicutes and Bacteroidota phyla. The COMB group was characterized by family Muribaculaceae (from the Bacteroidota phylum) and *Lachnospiraceae* NK4A136 *group* and *Tuzzerella* (from the Firmicutes phylum). When comparing the COMB group to the control (C0), the COMB group’s biomarkers belonged to the Clostridia class and the C0 group’s biomarkers belonged to the Bacilli class (Fig. 7d). T-tests further explored differences in microbiome composition across taxonomic levels, supporting findings from the LEfSe analysis. At the phylum level, the abundance of Firmicutes was significantly higher in the FMT group compared to both the control and the DSS and COMB groups, while the abundance of Bacteroidota was significantly lower in the FMT group (Fig. 8a). No significant differences were observed between the DSS group and either C0 or the COMB group at the phylum level. At the family level (Fig. 8b), a consistent pattern across all experimental groups compared to C0 included an increase in Lachnospiraceae and Oscillospiraceae and a decrease in Enterococcaceae, aligning with LEfSe results. Peptostreptococcaceae exhibited opposing trends, with increased abundance in the FMT group and decreased abundance in the DSS group after UC induction. Additionally, the *Clostridia vadinBB60 group* increased in both the DSS and COMB groups. When comparing induction methods, the FMT and COMB groups displayed higher levels of Lachnospiraceae compared to the DSS group. In contrast, the DSS group had the highest abundance of Bacteroidaceae, consistent with LEfSe results. Among the experimental groups, the DSS and COMB groups showed the fewest significant differences. Comparisons with the FMT group revealed similar patterns in both the DSS and COMB groups, including higher abundance of Muribaculaceae and the *Clostridia vadinBB60 group* and lower abundance of Enterococcaceae, Peptostreptococcaceae, and Erysipelotrichaceae. These results were also aligned with the LEfSe analysis.

## Discussion

Alterations in the gut microbiome are a hallmark of UC, yet existing experimental models rarely incorporate these microbial changes. To address this gap, we used faecal microbiota from a UC patient to induce colitis in mice and compared its effects with the widely used DSS model and a combination of these triggers, aiming to explore more translationally relevant approaches. Our results indicate that FMT alone had only a minimal impact on the overall DAI (Fig. 1), which remained low throughout the experiment. The significant but modest increase in DAI at the end of the induction period suggests that the five-day course used in this study, optimized for DSS administration, may have been insufficient for FMT to induce pronounced clinical symptoms. Based on published literature, a long-term colonization of germ-free mice is typically recommended for disease development [19]. In contrast, DSS induced a rapid escalation of DAI, more reminiscent of fulminant colitis than of the mild-to-moderate activity seen in most UC patients. Interestingly, the COMB protocol produced an intermediate, yet sustained increase in DAI that was significantly higher than in FMT and control animals but consistently lower than in the DSS group. This pattern indicates that the combination of UC microbiota with a chemical trigger generates a clinically evident but non-fulminant colitis, suggesting that the COMB model may better mirror the mild-to-moderate disease activity typically observed in human UC [20, 21] while simultaneously allowing the contribution of the dysbiotic microbiota to be studied. This aligns with previous studies demonstrating the protective role of the gut microbiome against DSS-induced colitis [22]. Analysis of immune cell populations in the colon mucosa and secondary lymphoid organs revealed distinct immune responses to each induction stimulus (Fig. 2). Nevertheless, all three experimental groups exhibited a pronounced inflammatory response, characterized by significant leukocyte and neutrophil infiltration into the colon. Such an inflammatory response is typical for UC [23, 24], and our results demonstrate that UC microbiota alone is sufficient to elicit mucosal inflammation. A comparable macrophage response was observed across all groups, marked by a surprising reduction in their population. We hypothesize that this reflects the behaviour of tissue-resident F4/80^hi^ CX3CR1^hi^ macrophages, which exert anti-inflammatory functions during homeostasis and are progressively replaced during inflammation by monocyte-derived macrophages that express intermediate levels of CX3CR1 [25] and often lower levels of F4/80. Both resident and inflammatory macrophages originate from circulating Ly6C^+^ monocytes, but under inflammatory conditions these cells preferentially differentiate into inflammatory macrophages, only some of which retain strong F4/80 expression [26, 27]. As a consequence, the F4/80^+^ compartment may not be efficiently replenished, leading to the apparent decrease observed in our experiments. Similar reductions in F4/80^+^ macrophages in inflamed colonic tissue, often accompanied by the accumulation of monocyte-derived inflammatory myeloid cells or by the redistribution of CD11b^+^ F4/80^+^ macrophages to draining lymphoid organs, have been reported in other murine colitis models [28], as well as by Bain et al., who described a trend towards reduced resident macrophages under inflammatory conditions [26]. Future studies combining F4/80 with CX3CR1 and additional markers will be required to more precisely dissect these subsets.

Variation in immune responses to different induction methods was particularly evident in the NK and NKT cell populations. Administration of FMT, either alone or in combination with DSS, resulted in a reduction of both NK and NKT cells, whereas DSS alone led to an increase in NKT cells. While the precise role of NK cells in IBD remains unclear, studies have reported that mice depleted of NK cells had increased neutrophils and rapidly succumb to DSS-induced colitis [29]. Our data showed a similar trend, with NK cells negatively correlating with neutrophils, suggesting that loss of NK cells may permit excessive neutrophil accumulation. On the other hand, regarding the NKT cell population, we employed a phenotypic definition (CD8^+^ CD49b^+^) based on the established gating strategy of Bayne et al. [18] While we acknowledge that this strategy does not utilize TCRβ to strictly segregate canonical NKT cells, the observed trends align closely with known microbiota-dependent NKT biology. The establishment of intestinal NKT cells has been proposed to be closely linked to the microbiota. Antibiotic-treated animals exhibit increased NKT cells [30], consistent with our findings, as both C0 and DSS groups showed higher NKT cell numbers. Similar to our DSS group, oxazolone-induced colitis has also been associated with elevated NKT cell response [31]. UC is often considered a Th2-skewed disease mediated in part by IL-13-producing NKT cells that contribute to epithelial damage [32]. However, NKT cells can be both protective and pathogenic, as studies in Jα18−/− mice have shown mixed outcomes [33, 34]. Our data therefore support the view that DSS predominantly triggers an acute, NKT-dominated innate response, which is in line with the well-established notion that acute DSS colitis can develop even in the absence of T and B lymphocytes and is therefore mainly driven by barrier damage and innate immune mechanisms rather than fully adaptive, T cell–dependent immunopathogenesis [35]. FMT and COMB shift the balance away from innate cytotoxic effectors and toward adaptive lymphocyte-driven inflammation. This is more reminiscent of the lymphoplasmacytic infiltrates that characterise human UC mucosa [36, 37], in which T and B cells and plasma cells represent the dominant immune populations, while neutrophils accumulate mainly during acute flares.

Regarding adaptive immunity, we analysed lymphocyte subpopulations in the colon mucosa, Peyer’s patches, and spleen. B cell infiltration and expansion under inflammatory conditions is well documented in Peyer’s patches [38] and the spleen [39], as well as in inflamed mucosa in UC [40]. In our study, DSS treatment was associated with lower relative frequencies of B cells in all three compartments compared with the other experimental groups, most prominently in Peyer’s patches and spleen. The observed decrease of relative B cell frequency in the colonic mucosa following DSS treatment is consistent with a recent study [41]. However, changes in all three analysed compartments represent a moderate decrease rather than a complete loss of B cells and indicate a myeloid-skewed, B-cell-poor pattern of inflammation. In the colon mucosa of antibiotic-conditioned pseudo–germ-free mice, this B-cell-poor profile likely reflects DSS-induced damage of an already fragile epithelial barrier, favouring recruitment of neutrophils and monocyte/macrophages over local B-cell expansion. This pattern is consistent with the concept that acute DSS colitis is primarily an epithelial toxicity – and innate immunity-driven model rather than a B cell-dependent adaptive colitis. The modest reduction in splenic B-cell frequencies in DSS-treated mice most likely reflects a relative expansion of myeloid populations rather than specific B-cell depletion [35, 42]. In contrast, mice receiving UC microbiota, either alone or in combination with DSS, displayed increased proportions of B lymphocytes in Peyer’s patches and spleen and preserved or slightly increased B cells in the colon compared with DSS. This B-cell-rich profile in gut-associated and systemic lymphoid tissues more closely resembles the chronic lymphoplasmacytic infiltrates described in human UC mucosa [36] than the DSS phenotype and suggests that UC microbiota is a dominant driver of B-cell responses even in the presence of a chemical insult. The COMB protocol therefore combines DSS-induced epithelial injury and clinical inflammation with a microbiota-driven, B-cell-rich immune landscape, providing a complementary, more UC-like context for studying the microbiota–B cell axis compared with DSS alone. Helper T cells increased significantly in Peyer’s patches across all groups and in the spleen only in DSS-treated mice, consistent with prior reports of Th-cell expansion during experimental colitis [43]. Although UC is often classified as Th2-driven disease [44], total Th cells frequencies in the colon remained unchanged. However, this points to a limitation of our study, as we did not analyse specific Th subpopulations, where functionally relevant shifts (e.g. Th1, Th17, Tfh, Treg) may have occurred. At the same time, our data underscore important differences between induction protocols at the CD8^+^ T-cell level. Stevceva et al. [45] reported no significant changes in colonic CD4+ and CD8+ T cells during DSS colitis, aligning with our DSS group. In contrast, increased CD8+ T cells have been reported in patients with active UC [46], a finding observed only in the COMB group, suggesting that the combined action of UC microbiota and epithelial injury is required to trigger a UC-like cytotoxic T-cell response in the colon, highlighting CD8^+^ T-cell infiltration as one of the key aspects of human UC pathophysiology [47] that is captured by the COMB model but not by DSS alone.

Transcriptomic analysis provided further insight into the inflammatory response and suggested distinct pathways through which DSS and FMT drive inflammation. Results from our study showed that the UC-derived microbiota induced more robust changes in the colon transcriptome, as evidenced by a higher number of DEGs in the FMT and COMB groups compared to the DSS group (Fig. 3c; Fig. 4). While the substantial impact of the gut microbiota on host gene expression has been well documented in previous studies [48], it is important to note that the relatively lower number of DEGs observed in the DSS group may be biased due to its higher intragroup variability (Fig. 3b). Despite this limitation, our analysis suggests that DSS and microbiota interact with the intestinal epithelial barrier in distinct ways. The impact of microbiota on the colon mucosa was most pronounced in tight junction and mucin-related gene expression (Figs. 4a, 4b) while DSS had a greater effect on the transcription of inflammation-related genes (Figs. 4c, 4d; Fig. 5). Defects in the intestinal epithelial barrier (IEB) have long been recognized as a hallmark of IBDs [49] and are considered a crucial factor in UC pathogenesis, with some researchers even proposing that IBDs are fundamentally a disease of the intestinal barrier [50]. Our findings suggest that the microbiome may be a key driver of these intestinal barrier dysregulations, as we observed significant downregulation of tight junction genes following UC FMT (Fig. 4a; Table 3; Additional files 5 and 6). Such downregulation has been reported in biopsies from patients with UC [51]. Interestingly, we also noted the downregulation of *Cldn2*, a ‘barrier-weakening’ claudin whose overexpression is typically associated with active UC [52]. Surprisingly, DSS treatment significantly altered the expression of only one tight junction gene, *Ocln*. In a study using the Caco-2 intestinal epithelium model, DSS was shown to redistribute tight junction proteins, leading to barrier dysfunction [53]. This suggests that while DSS disrupts the IEB primarily through post-translational mechanisms, such as protein redistribution, the microbiota may contribute to barrier dysfunction through transcriptional regulation. However, previous studies have reported downregulation of tight junction proteins in DSS-induced colitis models as well [54]. Similarly to tight junctions’ genes, microbiota appeared to have a greater influence on mucin expression (Fig. 4b; Table 4), and its effects may have been distinct from or even opposing to those of DSS. Downregulation of the mucin expression has been reported in patients with UC and CD [55] although findings regarding specifically *Muc2* expression remain conflicting [56]. On the other hand, DSS induced a stronger inflammatory response, as indicated by the upregulation of several key chemokines and cytokines (Figs. 4c and d; Table 5), many of which are known to be elevated in UC patients and implicated in disease pathogenesis [57, 58]. While the administration of UC microbiota alone had only a minimal effect on expression of inflammation-related genes, the combined effect of FMT and DSS resulted in the upregulation of even more genes than DSS alone, suggesting a potential synergistic interaction. Our analysis of the most significant DEGs further supports the observation that DSS administration induces a more severe inflammatory response. Among the top 10 most significant genes (Fig. 5b), several (*Gsdmc2, Gsdmc3,* and *Gsdmc4*) belonged to the gasdermin family. Gasdermins, particularly classes A and B, have been implicated in intestinal inflammation and IBD susceptibility [59] and recently it has been shown that gasdermin C genes are upregulated by type 2 immunity [60]. Other genes that were upregulated after DSS administration included *Ggt1*, an early marker of subclinical inflammation and oxidative stress [61]; *Mt1*, which has been reported to be significantly overexpressed in inflamed colon tissue from DSS-induced colitis models [62]; and *Krt7* which has been implicated in colorectal carcinoma [63]. Although *Krt7* was upregulated in the DSS group compared to the control group, it was found to be downregulated when compared to the COMB group (Fig. 5e), indicating that its expression was even higher in the COMB group. Compared to the FMT group (Fig. 5d), the DSS group also showed upregulation of *Ffar4* and *Calr*, whose increased expression has been observed in patients with UC [64, 65], as well as *Hspa8*, which has been associated with colorectal cancer [66]. Several inflammation-related genes were upregulated in the COMB group when compared to C0 (Fig. 5c), most notably *Osmr*, which is overexpressed in IBD lesions and correlates with disease severity [67]. Additionally, *Mmp3*, encoding coding matrix metalloproteinase 3, was upregulated. This enzyme enhances vascular permeability, facilitating neutrophil and monocyte infiltration at sites of tissue damage [68]. Genes for interferon-inducible transmembrane proteins *Ifitm1* and *Ifitm3* were also overexpressed. These genes are expressed by T cells and are directly involved in regulation of adaptive immunity [69]. However, their role in inflammation appears context-dependent as a study by Alteber et al. suggested their role in ameliorating colitis and colitis-associated tumorigenesis [70]. Untargeted transcriptomic analysis also revealed increased expression of *H19* in the COMB group. *H19* belongs to the family of long non-coding RNAs. In addition to studies confirming its upregulation in both patients and experimental models of UC [71, 72], there is evidence that its role in pathogenesis may be primarily related to its detrimental effect on the IEB [73]. Compared to the DSS group, *Slit2* was upregulated in the COMB group (Fig. 5e). Reduced expression of this gene has been observed in UC [74]. Several of the most significant DEGs in FMT group (Fig. 5a) have also been implicated in inflammation and UC pathogenesis. Notably, the downregulation of *Mptx* has been linked to increased susceptibility to inflammation and colitis [75] while *Meg3*, another downregulated gene, has been proposed to have a protective role in UC by alleviating inflammation [76]. One of the two upregulated genes in the FMT group was *Snhg11*, one of small nucleolar RNA non-coding host genes. Non-coding RNAs like *Snhg11* have been shown to affect the intestinal mucosal barrier, promoting the pathogenesis and progression of multiple diseases [77], and the overexpression of *Snhg11* has been implicated in colorectal carcinoma [78]. The notable role of UC FMT as an inductive agent in triggering experimental colitis, compared with DSS, was further supported by the finding that the DSS group showed increased expression of *Manf*, *Galnt12*, *Zg16*, and *Muc2* relative to the FMT group (Fig. 5d). Downregulation of these genes, as observed in the FMT group, has been associated with the infiltration of proinflammatory macrophages into the lamina propria [79], the development of colorectal carcinoma [80], or ulcerative colitis [81–83].

Microbiome analysis revealed a clear distinction between the effects of FMT and DSS as induction methods and showed that the COMB protocol largely superimposes DSS-induced diversity loss on a UC-like dysbiotic community. Decreased alpha diversity is a well-established hallmark of IBDs [84, 85], and DSS treatment replicates this feature [86], which was also evident in our study (Fig. 6a). Interestingly, the decline in microbial richness and evenness persisted when DSS was combined with FMT, indicating that DSS exerts a dominant, long-lasting detrimental effect on α-diversity even in the presence of transplanted microbiota. Previous studies have reported that FMT should counteract the detrimental effects of antibiotics [87] on species richness and evenness in a short time span [88]. In our study, however, UC-derived FMT restored only the Simpson index to pre-antibiotic levels, suggesting that the transplanted microbiota was itself dysbiotic and unable to fully reconstitute a healthy, diverse microbiota. Beta-diversity analyses further underscored these differences: hierarchical clustering and PCoA based on weighted UniFrac distances placed FMT and COMB communities together and clearly separated from C0/INT and DSS (Fig. 6b,d), indicating that combining DSS with FMT does not revert the microbiota to a DSS-like profile but maintains an FMT-derived community on a background of severe colitis. Microbiome biomarker analysis via LEfSe (Fig. 7), supported by t-tests (Fig. 8), confirmed that the FMT group displayed microbial signatures most closely resembling those described in IBD patients. A higher abundance of the phylum Proteobacteria, identified as a marker of the FMT group in our study, has been consistently reported in UC [89, 90]. Within this phylum, we detected several taxa that have been associated with intestinal inflammation, including class Gammaproteobacteria [91], family Morganellaceae [92], genus *Providencia* [93], and species *Proteus mirabilis* [94]. On the other hand, we also observed an increase in the phylum Firmicutes in the FMT group, which contradicts published evidence, as most studies report a decrease in Firmicutes [95, 96]. However, certain subtaxa within Firmicutes have been implicated in UC, including those reported in the FMT group in our study, such as class Clostridia [97], family Peptostreptococcaceae [98], and genus *Anaerotruncus* [99]. Additionally, we identified *Clostridioides difficile* (previously *Clostridium difficile*), as a biomarker in the FMT group which further supports the inflammatory potential of the transferred UC microbiota. Indeed, *C. difficile* infection is known to exacerbate UC, increasing disease severity, flare duration, and complication risk [100]. Together, these findings indicate that UC-derived FMT established a microbiota with multiple UC-like dysbiotic features rather than restoring a healthy community. On the other hand, the microbial changes observed in the DSS group mostly contradict published findings on the microbiome in IBD (Figs. 7a, 7c; Fig. 8). While DSS is known to promote gut microbiome dysbiosis [86], our results suggest that this dysbiosis primarily manifested as a reduction in alpha diversity (Fig. 6a), whereas the specific microbial compositional changes identified through LEfSe analysis and t-tests do not align with typical UC-associated patterns. For instance, we identified Bacteroidaceae, *Paludicola* and *Muribaculum*, and *Bacteroides caecimuris* as biomarkers of the DSS group, despite prior reports indicating a decrease in these taxa in UC [97, 101, 102] or their association with treatment response rather than active disease [103, 104]. Notably, families Lachnospiraceae and Oscillospiraceae emerged as biomarkers across all induction methods when compared to the control group, with their increased abundance further supported by t-test results. However, Lachnospiraceae has predominantly been reported as reduced in UC [105, 106], while a higher abundance of Oscillospiraceae has been linked to treatment response in IBD [107, 108]. That said, some evidence does support an increase in Lachnospiraceae [97] in UC. Thus, DSS alone primarily captures the loss of diversity but not the characteristic taxonomic configuration of UC. The COMB group did not generate a large number of unique biomarkers in LEfSe, but retained key elements of both triggers: DSS-like reductions in α-diversity and the FMT-derived UC-like taxa, as indicated by its clustering with FMT in beta-diversity analyses and by t-test comparisons (Figs. 6–8). From a translational perspective, FMT alone best recapitulates the compositional features of human UC dysbiosis, whereas the COMB protocol provides a context in which this dysbiotic, UC-derived microbiota is maintained on a background of severe DSS-induced colitis. This complements the DSS model, which mainly reflects non-specific diversity loss without a UC-like microbial signature, and supports the use of COMB when interventions targeting both inflammation and dysbiotic microbiota are to be evaluated.

A further aspect that was beyond the scope of the present work is a detailed analysis of donor engraftment. Although the human UC stool sample used for FMT preparation was not available with sufficient replication for group-level statistics, our 16S rRNA data still capture the overall community shifts in mice after exposure to UC FMT and/or DSS rather than providing a rigorous, taxon-resolved quantification of donor colonisation. A more in-depth characterisation of engraftment, including parallel profiling of donor material and recipient microbiota, longitudinal sampling, and an extended colonisation period, will be an important focus of future studies.

## Conclusions

In conclusion, this study compared chemical, microbial and combined induction strategies to model different facets of ulcerative colitis. DSS alone primarily reproduced acute, neutrophil-dominated colitis with the most pronounced clinical symptoms, upregulation of chemokine- and inflammation-related genes and a marked loss of microbial α-diversity, but with a B-cell–poor immune profile and a microbiome configuration that only partly resembled UC dysbiosis. UC microbiota transfer (FMT) established a dysbiotic community with microbial signatures consistent with those described in UC patients and induced robust transcriptional dysregulation of tight junction and mucin genes, together with B-cell expansion in gut-associated and systemic lymphoid tissues, but caused only mild clinical disease. The combined protocol (COMB) integrated key features of both approaches: it retained DSS-like clinical inflammation and diversity loss, while adding UC-like barrier-gene suppression, a B-cell-rich immune landscape and a UC-relevant cytotoxic CD8^+^ T-cell response, along with the broadest activation of UC-associated inflammation-related genes such as OSMR, MMP3, H19 and IFITM1/3. By engaging adaptive lymphocyte compartments on top of DSS-driven innate epithelial injury, COMB partially compensates for the lack of T- and B-cell dependence in classical acute DSS colitis and brings the immune-cell landscape of this acute model closer to that observed in human UC. Thus, COMB uniquely captures the interplay between dysbiotic microbiota, epithelial-barrier dysfunction and mucosal inflammation that is not achieved by DSS alone. Taken together, our data suggest that FMT is particularly suited for studying microbiome-driven mechanisms, whereas the COMB model provides a complementary, more UC-like context for testing interventions that target both the intestinal barrier and the dysbiotic microbiota within an acutely inflamed, lymphocyte-rich colon. Further refinement and validation of these microbiome-inclusive models will be essential to enhance their robustness and translational applicability.

## Electronic supplementary material

Below is the link to the electronic supplementary material.

Supplementary Material 1

Supplementary Material 2

Supplementary Material 3

Supplementary Material 4

Supplementary Material 5

Supplementary Material 6

Supplementary Material 7

## Data Availability

The RNA-seq datasets generated and analysed during the current study are available in the Gene Expression Omnibus (GEO) repository under accession number GSE305760. The 16S rRNA sequencing datasets are available in the Sequence Read Archive (SRA) under accession number PRJNA1307408.

## Abbreviations

ACE: Abundance-based coverage estimator
CD: Crohn’s disease
DAI: Disease activity index
DEGs: Differentially expressed genes
DSS: Dextran sodium sulphate
FDR: False discovery rate
FMT: Faecal microbiota transplantation
GIT: Gastrointestinal tract
HBSS: Hank’s balanced salt solution
HCA: Hierarchical cluster analysis
IBD: Inflammatory bowel disease
IEB: Intestinal epithelial barrier
KEGG: Kyoto encyclopaedia of genes and genomes
LEfSe: Linear discriminant analysis effect size
MRPP: Multi-response permutation procedure
NK: Natural killer
PCA: Principal component analysis
PCoA: Principal coordinate analysis
SPF: Specific pathogen-free
Tc: Cytotoxic T cells
Th: Helper T cells
TNBS: Trinitrobenzene sulfonic acid
UC: Ulcerative colitis
UPGMA: Unweighted pair group method with arithmetic mean

## References

[1] Mak WY, Zhao M, Ng SC, Burisch J. The epidemiology of inflammatory bowel disease: east meets west. J Gastro Hepatol. 2020;35(3):380–89. 10.1111/jgh.14872. 10.1111/jgh.14872 31596960

[2] Kaenkumchorn T, Wahbeh G. Ulcerative colitis: making the diagnosis. Gastroenterol Clin N Am. 2020;49(4):655–69. 10.1016/j.gtc.2020.07.001. 10.1016/j.gtc.2020.07.001 33121687

[3] Porter RJ, Kalla R, Ho GT. Ulcerative colitis: recent advances in the understanding of disease pathogenesis. F1000Res. 2020;9: F1000 Faculty Rev-294. 10.12688/f1000research.20805.1. 32399194 10.12688/f1000research.20805.1 PMC7194476

[4] Perše M, Cerar A. Dextran sodium sulphate colitis mouse model: traps and tricks. J Biomed Biotechnol. 2012;2012:1–13. 10.1155/2012/718617. 22665990 10.1155/2012/718617 PMC3361365

[5] Katsandegwaza B, Horsnell W, Smith K. Inflammatory bowel disease: a review of pre-clinical murine models of human disease. Int J Mol Sci. 2022;23(16):9344. 10.3390/ijms23169344. 36012618 10.3390/ijms23169344 PMC9409205

[6] Chieppa M, De Santis S, Verna G. Winnie mice: a chronic and progressive model of ulcerative colitis. Inflamm Bowel Dis. 2025;31(4):1158–67. 10.1093/ibd/izaf006. 39912845 10.1093/ibd/izaf006 PMC11985403

[7] Mizoguchi A, Takeuchi T, Himuro H, Okada T, Mizoguchi E. Genetically engineered mouse models for studying inflammatory bowel disease. J Pathol. 2016;238(2):205–19. 10.1002/path.4640. 26387641 10.1002/path.4640 PMC4689626

[8] Zakerska-Banaszak O, Tomczak H, Gabryel M, Baturo A, Wolko L, Michalak M, et al. Dysbiosis of gut microbiota in Polish patients with ulcerative colitis: a pilot study. Sci Rep. 2021;11(1):2166. 10.1038/s41598-021-81628-3. 33495479 10.1038/s41598-021-81628-3 PMC7835370

[9] Halfvarson J, Brislawn CJ, Lamendella R, Vázquez-Baeza Y, Walters WA, Bramer LM, et al. Dynamics of the human gut microbiome in inflammatory bowel disease. Nat Microbiol. 2017;2(5):17004. 10.1038/nmicrobiol.2017.4. 28191884 10.1038/nmicrobiol.2017.4 PMC5319707

[10] Laryushina Y, Samoilova-Bedych N, Turgunova L, Kozhakhmetov S, Alina A, Suieubayev M, et al. Alterations of the gut microbiome and TMAO levels in patients with ulcerative colitis. J Clin Med. 2024;13(19):5794. 10.3390/jcm13195794. 39407853 10.3390/jcm13195794 PMC11477140

[11] Kedia S, Ghosh TS, Jain S, Desigamani A, Kumar A, Gupta V, et al. Gut microbiome diversity in acute severe colitis is distinct from mild to moderate ulcerative colitis. J Gastro Hepatol. 2021;36(3):731–39. 10.1111/jgh.15232. 10.1111/jgh.15232 32870508

[12] Shah R, Cope JL, Nagy-Szakal D, Dowd S, Versalovic J, Hollister EB, et al. Composition and function of the pediatric colonic mucosal microbiome in untreated patients with ulcerative colitis. Gut Microbes. 2016;7(5):384–96. 10.1080/19490976.2016.1190073. 27217061 10.1080/19490976.2016.1190073 PMC5046168

[13] Low D, Nguyen DD, Mizoguchi E. Animal models of ulcerative colitis and their application in drug research. Drug Des Devel Ther. 2013;7:1341–57. 24250223 10.2147/DDDT.S40107 PMC3829622

[14] Cahill RJ, Foltz CJ, Fox JG, Dangler CA, Powrie F, Schauer DB. Inflammatory bowel disease: an immunity-mediated condition triggered by bacterial infection with *Helicobacter hepaticus*. Infect Immun. 1997;65(8):3126–31. 10.1128/iai.65.8.3126-3131.1997. 9234764 10.1128/iai.65.8.3126-3131.1997 PMC175441

[15] Popper M, Gancarčíková S, Maďar M, Mudroňová D, Hrčková G, Nemcová R. Amoxicillin-clavulanic acid and ciprofloxacin-treated SPF mice as gnotobiotic model. Appl Microbiol Biotechnol. 2016;100(22):9671–82. 10.1007/s00253-016-7855-3. 27695915 10.1007/s00253-016-7855-3

[16] Lauko S, Gancarcikova S, Hrckova G, Hajduckova V, Andrejcakova Z, Fecskeova LK, et al. Beneficial effect of faecal microbiota transplantation on mild, moderate and severe dextran sodium sulphate-induced ulcerative colitis in a pseudo germ-free Animal model. Biomedicines. 2023;12(1):43. 10.3390/biomedicines12010043. 38255150 10.3390/biomedicines12010043 PMC10813722

[17] Gancarcikova S, Lauko S, Hrckova G, Andrejcakova Z, Hajduckova V, Madar M, et al. Innovative animal model of DSS-Induced ulcerative colitis in pseudo germ-free mice. Cells. 2020;9(12):2571. 10.3390/cells9122571. 33271873 10.3390/cells9122571 PMC7761014

[18] Bayne LJ, Vonderheide RH. Multicolor flow cytometric analysis of immune cell subsets in tumor-bearing mice. Cold Spring Harb Protoc. 2013;2013(10):955–60. 10.1101/pdb.prot077198. 24086051 10.1101/pdb.prot077198

[19] Jans M, Vereecke L. A guide to germ-free and gnotobiotic mouse technology to study health and disease. The FEBS J. 2025;292(6):1228–51. 10.1111/febs.17124. 38523409 10.1111/febs.17124

[20] Singh S, Feuerstein JD, Binion DG, Tremaine WJ. AGA technical review on the management of mild-to-moderate ulcerative colitis. Gastroenterology. 2019;156(3):769–808.e29. 10.1053/j.gastro.2018.12.008. 30576642 10.1053/j.gastro.2018.12.008 PMC6858923

[21] Rodríguez-Lago I, Barreiro-de Acosta M. Mild to moderate ulcerative colitis: call me by my name. U Eur Gastroenterol J. 2022;10(9):919–20. 10.1002/ueg2.12299. 10.1002/ueg2.12299 PMC9731657 36031778

[22] Yang Y, He J, Wang Y, Liang L, Zhang Z, Tan X, et al. Whole intestinal microbiota transplantation is more effective than fecal microbiota transplantation in reducing the susceptibility of DSS-induced germ-free mice colitis. Front Immunol. 2023;14:1143526. 37234168 10.3389/fimmu.2023.1143526 PMC10206398

[23] Kvedaraite E, Lourda M, Ideström M, Chen P, Olsson-Åkefeldt S, Forkel M, et al. Tissue-infiltrating neutrophils represent the main source of IL-23 in the colon of patients with IBD. Gut. 2016;65(10):1632–41. 10.1136/gutjnl-2014-309014. 26160381 10.1136/gutjnl-2014-309014

[24] Maruta K, Watanabe C, Hozumi H, Kurihara C, Furuhashi H, Takajo T, et al. Nicotine treatment ameliorates DSS-induced colitis by suppressing MAdCAM-1 expression and leukocyte recruitment. J Leukoc Biol. 2018;104(5):1013–22. 29901817 10.1002/JLB.3A0717-304R

[25] Cerovic V, Bain CC, Mowat AM, Milling SWF. Intestinal macrophages and dendritic cells: what’s the difference? Trends Immunol. 2014;35(6):270–77. 10.1016/j.it.2014.04.003. 24794393 10.1016/j.it.2014.04.003

[26] Bain CC, Scott CL, Uronen-Hansson H, Gudjonsson S, Jansson O, Grip O, et al. Resident and pro-inflammatory macrophages in the colon represent alternative context-dependent fates of the same Ly6Chi monocyte precursors. Mucosal Immunol. 2013;6(3):498–510. 22990622 10.1038/mi.2012.89 PMC3629381

[27] Zigmond E, Varol C, Farache J, Elmaliah E, Satpathy AT, Friedlander G, et al. Ly6Chi monocytes in the inflamed colon give rise to proinflammatory effector cells and migratory antigen-presenting cells. Immunity. 2012;37(6):1076–90. 10.1016/j.immuni.2012.08.026. 23219392 10.1016/j.immuni.2012.08.026

[28] Corbin AL, Gomez-Vazquez M, Berthold DL, Attar M, Arnold IC, Powrie FM, et al. IRF5 guides monocytes toward an inflammatory CD11c+ macrophage phenotype and promotes intestinal inflammation. Sci Immunol. 2020;5(47):eaax 6085. 10.1126/sciimmunol.aax6085 PMC7611075 32444476

[29] Hall LJ, Murphy CT, Quinlan A, Hurley G, Shanahan F, Nally K, et al. Natural killer cells protect mice from DSS-induced colitis by regulating neutrophil function via the NKG2A receptor. Mucosal Immunol. 2013;6(5):1016–26. 23340823 10.1038/mi.2012.140

[30] Burrello C, Garavaglia F, Cribiù FM, Ercoli G, Bosari S, Caprioli F, et al. Short-term oral antibiotics treatment promotes inflammatory activation of colonic invariant natural killer T and conventional CD4+ T cells. Front Med (Lausanne). 2018;5:21. 29468162 10.3389/fmed.2018.00021 PMC5808298

[31] Heller F, Fuss IJ, Nieuwenhuis EE, Blumberg RS, Strober W. Oxazolone colitis, a Th2 colitis model resembling ulcerative colitis, is mediated by IL-13-producing NK-T cells. Immunity. 2002;17(5):629–38. 10.1016/S1074-7613(02)00453-3. 12433369 10.1016/s1074-7613(02)00453-3

[32] Fuss IJ, Strober W. The role of IL-13 and NK T cells in experimental and human ulcerative colitis. Mucosal Immunol. 2008;1(1):S31–3. 10.1038/mi.2008.40. 10.1038/mi.2008.40 PMC3673706 19079225

[33] Shen S, Prame Kumar K, Stanley D, Moore RJ, Van TTH, Wen SW, et al. Invariant natural killer T cells shape the gut microbiota and regulate neutrophil recruitment and function during intestinal inflammation. Front Immunol. 2018;9:999. 29867976 10.3389/fimmu.2018.00999 PMC5949322

[34] Kim HS, Chung DH. IL-9-producing invariant NKT cells protect against DSS-induced colitis in an IL-4-dependent manner. Mucosal Immunol. 2013;6(2):347–57. 10.1038/mi.2012.77. 22892939 10.1038/mi.2012.77

[35] Chassaing B, Aitken JD, Malleshappa M, Vijay-Kumar M. Dextran sulfate sodium (DSS)-induced colitis in mice. Curr Protoc Immunol. 2014;104(1):.15.25.1–15.25.14. 10.1002/0471142735.im1525s104. 10.1002/0471142735.im1525s104 PMC3980572 24510619

[36] Magro F, Langner C, Driessen A, Ensari A, Geboes K, Mantzaris GJ, et al. European consensus on the histopathology of inflammatory bowel disease. J Crohns Colitis. 2013;7(10):827–51. 23870728 10.1016/j.crohns.2013.06.001

[37] DeRoche TC, Xiao SY, Liu X. Histological evaluation in ulcerative colitis. Gastroenterol Rep (Oxford). 2014;2(3):178–92. 10.1093/gastro/gou031. 10.1093/gastro/gou031 PMC4124271 24942757

[38] Burger C, Vitetta ES. The response of B cells in spleen, Peyer’s patches, and lymph nodes to LPS and IL-4. Cell Immunol. 1991;138(1):35–43. 10.1016/0008-8749(91)90130-4. 1913841 10.1016/0008-8749(91)90130-4

[39] Rauch PJ, Chudnovskiy A, Robbins CS, Weber GF, Etzrodt M, Hilgendorf I, et al. Innate response activator B cells protect against microbial sepsis. Science. 2012;335(6068):597–601. 10.1126/science.1215173. 22245738 10.1126/science.1215173 PMC3279743

[40] Polese L, Boetto R, De Franchis G, Angriman I, Porzionato A, Norberto L, et al. B1a lymphocytes in the rectal mucosa of ulcerative colitis patients. World J Gastroenterol. 2012;18(2):144–49. 22253520 10.3748/wjg.v18.i2.144 PMC3257441

[41] Galván-Peña S, Zhu Y, Hanna BS, Mathis D, Benoist C. A dynamic atlas of immunocyte migration from the gut. Sci Immunol. 2024;9(91):eadi 0672. 10.1126/sciimmunol.adi0672. 10.1126/sciimmunol.adi0672 PMC10964343 38181094

[42] Lee CH, Koh SJ, Radi ZA, Habtezion A. Animal models of inflammatory bowel disease: novel experiments for revealing pathogenesis of colitis, fibrosis, and colitis-associated colon cancer. Intest Res. 2023;21(3):295–305. 10.5217/ir.2023.00029. 37248173 10.5217/ir.2023.00029 PMC10397556

[43] Xu X, Wang Y, Zhang B, Lan X, Lu S, Sun P, et al. Treatment of experimental colitis by endometrial regenerative cells through regulation of B lymphocytes in mice. STEM Cell Res Ther. 2018;9(1):146. 10.1186/s13287-018-0874-5. 29784012 10.1186/s13287-018-0874-5 PMC5963178

[44] Roda G, Marocchi M, Sartini A, Roda E. Cytokine networks in ulcerative colitis. Ulcers. 2011;391787:1–5. 10.1155/2011/391787.

[45] Stevceva L, Pavli P, Husband AJ, Doe WF. The inflammatory infiltrate in the acute stage of the dextran sulphate sodium induced colitis: B cell response differs depending on the percentage of DSS used to induce it. BMC Clin Pathol. 2001;1(1):3. 10.1186/1472-6890-1-3. 11580872 10.1186/1472-6890-1-3 PMC57007

[46] Hone Lopez S, Kats-Ugurlu G, Renken RJ, Buikema HJ, de Groot MR, Visschedijk MC, et al. Immune checkpoint inhibitor treatment induces colitis with heavy infiltration of CD8 + T cells and an infiltration pattern that resembles ulcerative colitis. Virchows Arch. 2021;479(6):1119–29. 10.1007/s00428-021-03170-x. 34338882 10.1007/s00428-021-03170-x PMC8724151

[47] Casalegno Garduño R, Däbritz J. New insights on CD8+ T cells in inflammatory bowel disease and Therapeutic approaches. Front Immunol. 2021;12:738762. 10.3389/fimmu.2021.738762. 34707610 10.3389/fimmu.2021.738762 PMC8542854

[48] Nichols RG, Davenport ER. The relationship between the gut microbiome and host gene expression: a review. Hum Genet. 2021;140(5):747–60. 10.1007/s00439-020-02237-0. 33221945 10.1007/s00439-020-02237-0 PMC7680557

[49] Schmitz H, Barmeyer C, Fromm M, Runkel N, Foss HD, Bentzel CJ, et al. Altered tight junction structure contributes to the impaired epithelial barrier function in ulcerative colitis. Gastroenterology. 1999;116(2):301–09. 10.1016/S0016-5085(99)70126-5. 9922310 10.1016/s0016-5085(99)70126-5

[50] Jäger S, Stange EF, Wehkamp J. Inflammatory bowel disease: an impaired barrier disease. Langenbecks Arch Surg. 2013;398(1):1–12. 10.1007/s00423-012-1030-9. 23160753 10.1007/s00423-012-1030-9

[51] Tan Y, Guan Y, Sun Y, Zheng C. Correlation of intestinal mucosal healing and tight junction protein expression in ulcerative colitis patients. Am J Med Ci. 2019;357(3):195–204. 10.1016/j.amjms.2018.11.011. 10.1016/j.amjms.2018.11.011 30638599

[52] Das P, Goswami P, Das TK, Nag T, Sreenivas V, Ahuja V, et al. Comparative tight junction protein expressions in colonic Crohn’s disease, ulcerative colitis, and tuberculosis: a new perspective. Virchows Arch. 2012;460(3):261–70. 10.1007/s00428-012-1195-1. 22297703 10.1007/s00428-012-1195-1

[53] Samak G, Chaudhry KK, Gangwar R, Narayanan D, Jaggar JH, Rao R. Calcium/Ask1/MKK7/JNK2/c-Src signalling cascade mediates disruption of intestinal epithelial tight junctions by dextran sulfate sodium. Biochem J. 2015;465(3):503–15. 10.1042/BJ20140450. 25377781 10.1042/BJ20140450 PMC4385020

[54] Poritz LS, Garver KI, Green C, Fitzpatrick L, Ruggiero F, Koltun WA. Loss of the tight junction protein ZO-1 in dextran sulfate sodium induced colitis. J Surg Res. 2007;140(1):12–19. 10.1016/j.jss.2006.07.050. 17418867 10.1016/j.jss.2006.07.050

[55] Dorofeyev AE, Vasilenko IV, Rassokhina OA, Kondratiuk RB. Mucosal barrier in ulcerative colitis and Crohn’s disease. Gastroenterol Res Pract. 2013;2013:431231. 23737764 10.1155/2013/431231 PMC3664489

[56] Bankole E, Read E, Curtis MA, Neves JF, Garnett JA. The relationship between Mucins and ulcerative colitis: a Systematic review. J Clin Med. 2021;10(9):1935. 10.3390/jcm10091935. 33946184 10.3390/jcm10091935 PMC8125602

[57] Trivedi PJ, Adams DH. Chemokines and chemokine receptors as therapeutic targets in inflammatory bowel disease; pitfalls and promise. J Crohns Colitis. 2018;12(suppl_2):S641–52. 10.1093/ecco-jcc/jjx145. 10.1093/ecco-jcc/jjx145 PMC6104621 30137309

[58] Neurath MF. Strategies for targeting cytokines in inflammatory bowel disease. Nat Rev Immunol. 2024;24(8):559–76. 10.1038/s41577-024-01008-6. 38486124 10.1038/s41577-024-01008-6

[59] Privitera G, Rana N, Armuzzi A, Pizarro TT. The gasdermin protein family: emerging roles in gastrointestinal health and disease. Nat Rev Gastroenterol Hepatol. 2023;20(6):366–87. 10.1038/s41575-023-00743-w. 36781958 10.1038/s41575-023-00743-w PMC10238632

[60] Gámez-Belmonte R, Wagner Y, Mahapatro M, Wang R, Erkert L, González-Acera M, et al. Intestinal epithelial gasdermin C is induced by IL-4R/STAT6 signaling but is dispensable for gut immune homeostasis. Sci Rep. 2024;14(1):26522. 10.1038/s41598-024-78336-z. 39489845 10.1038/s41598-024-78336-z PMC11532336

[61] Bo S, Gambino R, Durazzo M, Guidi S, Tiozzo E, Ghione F, et al. Associations between γ-glutamyl transferase, metabolic abnormalities and inflammation in healthy subjects from a population-based cohort: a possible implication for oxidative stress. World J Gastroenterol. 2005;11(45):7109–17. 10.3748/wjg.v11.i45.7109. 16437656 10.3748/wjg.v11.i45.7109 PMC4725082

[62] Dai H, Wang L, Li L, Huang Z, Ye YL. Metallothionein 1: a new spotlight on inflammatory diseases. Front Immunol. 2021;12:739918. 10.3389/fimmu.2021.739918. 34804020 10.3389/fimmu.2021.739918 PMC8602684

[63] Chen S, Su T, Zhang Y, Lee A, He J, Ge Q, et al. *Fusobacterium nucleatum* promotes colorectal cancer metastasis by modulating *KRT7-AS*/KRT7. Gut Microbes. 2020;11(3):511–25. 10.1080/19490976.2019.1695494. 31910722 10.1080/19490976.2019.1695494 PMC7524269

[64] Rodrigues BL, Dotti I, Pascoal LB, Morari J, Esteller M, Coope A, et al. Endoplasmic reticulum stress in colonic mucosa of ulcerative colitis patients is mediated by PERK and IRE1 pathway activation. Mediators Inflamm. 2022;2022:6049500. 35185383 10.1155/2022/6049500 PMC8849912

[65] Spathakis M, Dovrolis N, Filidou E, Kandilogiannakis L, Tarapatzi G, Valatas V, et al. Exploring microbial metabolite receptors in inflammatory bowel disease: an in silico analysis of their potential role in inflammation and fibrosis. Pharmaceuticals (Basel). 2024;17(4):492. 10.3390/ph17040492. 38675452 10.3390/ph17040492 PMC11054721

[66] Gemoll T, Kollbeck SL, Karstens KF, Hò GG, Hartwig S, Strohkamp S, et al. EB1 protein alteration characterizes sporadic but not ulcerative colitis associated colorectal cancer. Oncotarget. 2017;8(33):54939–50. 10.18632/oncotarget.18978. 28903393 10.18632/oncotarget.18978 PMC5589632

[67] West NR, Hegazy AN, Owens BMJ, Bullers SJ, Linggi B, Buonocore S, et al. Oncostatin M drives intestinal inflammation and predicts response to tumor necrosis factor–neutralizing therapy in patients with inflammatory bowel disease. Nat Med. 2017;23(5):579–89. 10.1038/nm.4307. 28368383 10.1038/nm.4307 PMC5420447

[68] Fingleton B. Matrix metalloproteinases as regulators of inflammatory processes. Biochim Biophys Acta Mol Cell Res. 2017;1864(11):2036–42. 10.1016/j.bbamcr.2017.05.010. 28502592 10.1016/j.bbamcr.2017.05.010

[69] Yánez DC, Ross S, Crompton T. The IFITM protein family in adaptive immunity. Immunology. 2020;159(4):365–72. 10.1111/imm.13163. 31792954 10.1111/imm.13163 PMC7078001

[70] Alteber Z, Sharbi-Yunger A, Pevsner-Fischer M, Blat D, Roitman L, Tzehoval E, et al. The anti-inflammatory IFITM genes ameliorate colitis and partially protect from tumorigenesis by changing immunity and microbiota. Immunol Cell Biol. 2018;96(3):284–97. 10.1111/imcb.12000. 29356071 10.1111/imcb.12000

[71] Geng H, Bu H-F, Liu F, Wu L, Pfeifer K, Chou PM, et al. In inflamed intestinal tissues and epithelial cells, interleukin 22 signaling increases expression of H19 long Noncoding RNA, which promotes mucosal regeneration. Gastroenterology. 2018;155(1):144–55. 10.1053/j.gastro.2018.03.058. 29621481 10.1053/j.gastro.2018.03.058 PMC6475625

[72] Yin L, Yan J, Chen W. Mechanism of lncRNA-H19 in intestinal injury of mice with ulcerative colitis. Int Arch Allergy Immunol. 2022;183(9):985–96. 10.1159/000524156. 35483327 10.1159/000524156

[73] Chen SW, Wang PY, Liu YC, Sun L, Zhu J, Zuo S, et al. Effect of long Noncoding RNA H19 overexpression on intestinal barrier function and its potential role in the pathogenesis of ulcerative colitis. Inflamm Bowel Dis. 2016;22(11):2582–92. 27661667 10.1097/MIB.0000000000000932

[74] Xie J, Li L, Deng S, Chen J, Gu Q, Su H, et al. Slit2/Robo1 mitigates DSS-induced ulcerative colitis by activating autophagy in intestinal stem cell. Int J Biol Sci. 2020;16(11):1876–87. 32398956 10.7150/ijbs.42331 PMC7211176

[75] Yan W, Chen S, Wang Y, You Y, Lu Y, Wang W, et al. Loss of Mptx2 alters bacteria composition and intestinal homeostasis potentially by impairing autophagy. Commun Biol. 2024;7(1):94. 10.1038/s42003-024-05785-7. 38218976 10.1038/s42003-024-05785-7 PMC10787791

[76] Wang YX, Lin C, Cui LJ, Deng TZ, Li QM, Chen FY, et al. Mechanism of M2 macrophage-derived extracellular vesicles carrying lncRNA MEG3 in inflammatory responses in ulcerative colitis. Bioengineered. 2021;12(2):12722–39. 10.1080/21655979.2021.2010368. 34895044 10.1080/21655979.2021.2010368 PMC8810016

[77] Yang T, Shen J. Small nucleolar RNAs and SNHGs in the intestinal mucosal barrier: emerging insights and current roles. J Adv Res. 2023;46:75–85. 10.1016/j.jare.2022.06.004. 35700920 10.1016/j.jare.2022.06.004 PMC10105082

[78] Wu Y. SNHG11: a new budding star in tumors and inflammatory Diseases. Mini Rev Med Chem. 2023;23(20):1993–2006. 10.2174/1389557523666230509122402. 37165588 10.2174/1389557523666230509122402

[79] Yang L, Shen W, Shao W, Zhao Q, Pang G, Yang Y, et al. MANF ameliorates DSS-induced mouse colitis via restricting Ly6ChiCX3CR1int macrophage transformation and suppressing CHOP-BATF2 signaling pathway. Acta Pharmacol Sin. 2023;44(6):1175–90. 10.1038/s41401-022-01045-8. 36635421 10.1038/s41401-022-01045-8 PMC10202914

[80] Meng H, Li W, Boardman LA, Wang L. Loss of ZG16 is associated with molecular and clinicopathological phenotypes of colorectal cancer. BMC Cancer. 2018;18(1):433. 10.1186/s12885-018-4337-2. 29661177 10.1186/s12885-018-4337-2 PMC5902988

[81] Dotti I, Mora-Buch R, Ferrer-Picón E, Planell N, Jung P, Masamunt MC, et al. Alterations in the epithelial stem cell compartment could contribute to permanent changes in the mucosa of patients with ulcerative colitis. Gut. 2017;66(12):2069–79. 10.1136/gutjnl-2016-312609. 27803115 10.1136/gutjnl-2016-312609 PMC5749340

[82] Lu Y, Su Y, Wang N, Li D, Zhang H, Xu H. Identification of O-glycosylation related genes and subtypes in ulcerative colitis based on machine learning. PLoS One. 2024;19(12):e0311495. 10.1371/journal.pone.0311495. 10.1371/journal.pone.0311495 PMC11687659 39739658

[83] Tytgat KMAJ, van der Wal J-W, Einerhand AWC, Büller HA, Dekker J. Quantitative analysis of MUC2 synthesis in ulcerative colitis. Biochem Biophys Res Commun. 1996;224(2):397–405. 8702401 10.1006/bbrc.1996.1039

[84] Zuo W, Wang B, Bai X, Luan Y, Fan Y, Michail S, et al. 16S rRNA and metagenomic shotgun sequencing data revealed consistent patterns of gut microbiome signature in pediatric ulcerative colitis. Sci Rep. 2022;12(1):6421. 10.1038/s41598-022-07995-7. 35440670 10.1038/s41598-022-07995-7 PMC9018687

[85] Rimmer P, Scott G, Quraishi MN, Hazel K, Cooney R, Hold G, et al. P107 is gut microbiome diversity important at IBD onset? A systematic review of the literature and meta-analysis of alpha diversity data. Gut. 2024;73(suppl_1):A114–15.

[86] Munyaka PM, Rabbi MF, Khafipour E, Ghia J. Acute dextran sulfate sodium (DSS)-induced colitis promotes gut microbial dysbiosis in mice. J Basic Microbiol. 2016;56(9):986–98. 10.1002/jobm.201500726. 27112251 10.1002/jobm.201500726

[87] Dethlefsen L, Huse S, Sogin ML, Relman DA. The pervasive effects of an antibiotic on the human gut microbiota, as revealed by deep 16S rRNA sequencing. PLoS Biol. 2008;6(11):e280. 10.1371/journal.pbio.0060280. 19018661 10.1371/journal.pbio.0060280 PMC2586385

[88] Le Bastard Q, Ward T, Sidiropoulos D, Hillmann BM, Chun CL, Sadowsky MJ, et al. Fecal microbiota transplantation reverses antibiotic and chemotherapy-induced gut dysbiosis in mice. Sci Rep. 2018;8(1):6219. 10.1038/s41598-018-24342-x. 29670191 10.1038/s41598-018-24342-x PMC5906603

[89] Zhou Y, Xu ZZ, He Y, Yang Y, Liu L, Lin Q, et al. Gut microbiota offers universal biomarkers across ethnicity in inflammatory bowel disease diagnosis and infliximab response prediction. mSystems. 2018;3(1):e00188–17. 10.1128/msystems.00188-17. 10.1128/mSystems.00188-17 PMC5790872 29404425

[90] Zhu S, Han M, Liu S, Fan L, Shi H, Li P. Composition and diverse differences of intestinal microbiota in ulcerative colitis patients. Front Cell Infect Microbiol. 2022;12:953962. 10.3389/fcimb.2022.953962. 36111238 10.3389/fcimb.2022.953962 PMC9468541

[91] Xu N, Bai X, Cao X, Yue W, Jiang W, Yu Z. Changes in intestinal microbiota and correlation with TLRs in ulcerative colitis in the coastal area of northern China. Microb Pathog. 2021;150:104707. 10.1016/j.micpath.2020.104707. 33352216 10.1016/j.micpath.2020.104707

[92] Dahal RH, Kim S, Kim YK, Kim ES, Kim J. Insight into gut dysbiosis of patients with inflammatory bowel disease and ischemic colitis. Front Microbiol. 2023;14:1174832. 10.3389/fmicb.2023.1174832. 37250025 10.3389/fmicb.2023.1174832 PMC10211348

[93] Neut C, Guillemot F, Colombel JF. Nitrate-reducing bacteria in diversion colitis: a clue to inflammation? Dig Dis Sci. 1997;42(12):2577–80. 10.1023/A:1018885217154. 9440640 10.1023/a:1018885217154

[94] Zhang J, Hoedt EC, Liu Q, Berendsen E, Teh JJ, Hamilton A, et al. Elucidation of Proteus mirabilis as a key bacterium in Crohn’s disease inflammation. Gastroenterology. 2021;160(1):317–30.e11. 33011176 10.1053/j.gastro.2020.09.036

[95] Tsai YC, Tai WC, Liang CM, Wu CK, Tsai MC, Hu WH, et al. Alternations of the gut microbiota and the Firmicutes/Bacteroidetes ratio after biologic treatment in inflammatory bowel disease. J Microbiol Immunol Infect. 2025;58(1):62–69. 39393964 10.1016/j.jmii.2024.09.006

[96] Tang Y, Liu H, Song G, Wu T, Zhao Y, Shi L. A case-control study on the association of intestinal flora with ulcerative colitis. AMB Express. 2021;11(1):106. 10.1186/s13568-021-01267-9. 34264407 10.1186/s13568-021-01267-9 PMC8282830

[97] Alam MT, Amos GCA, Murphy ARJ, Murch S, Wellington EMH, Arasaradnam RP. Microbial imbalance in inflammatory bowel disease patients at different taxonomic levels. Gut Pathog. 2020;12(1):1. 10.1186/s13099-019-0341-6. 31911822 10.1186/s13099-019-0341-6 PMC6942256

[98] Pittayanon R, Lau JT, Leontiadis GI, Tse F, Yuan Y, Surette M, et al. Differences in gut microbiota in patients with vs without inflammatory bowel diseases: a systematic review. Gastroenterology. 2020;158(4):930–46.e1. 31812509 10.1053/j.gastro.2019.11.294

[99] Vestergaard MV, Allin KH, Eriksen C, Zakerska-Banaszak O, Arasaradnam RP, Alam MT, et al. Gut microbiota signatures in inflammatory bowel disease. U Eur Gastroenterol J. 2024;12(1):22–33. 10.1002/ueg2.12485 PMC10859715 38041519

[100] Negrón ME, Rezaie A, Barkema HW, Rioux K, De Buck J, Checkley S, et al. Ulcerative colitis patients with *Clostridium difficile* are at increased risk of death, colectomy, and postoperative complications: a population-based inception cohort study. Am J Gastroenterol. 2016;111(5):691–704. 27091322 10.1038/ajg.2016.106

[101] Xu HM, Huang HL, Liu YD, Zhu JQ, Zhou YL, Chen HT, et al. Selection strategy of dextran sulfate sodium-induced acute or chronic colitis mouse models based on gut microbial profile. BMC Microbiol. 2021;21(1):279. 10.1186/s12866-021-02342-8. 34654370 10.1186/s12866-021-02342-8 PMC8520286

[102] Zhao H, Hu X, Guan S, Cai J, Li W, Zhang D, et al. Capilliposide a alleviates DSS-induced colitis by regulating the intestinal flora and its metabolites of origin. Int Immunopharmacol. 2025;146:113858. 39708482 10.1016/j.intimp.2024.113858

[103] Yao Q, Fan L, Zheng N, Blecker C, Delcenserie V, Li H, et al. 2′-fucosyllactose ameliorates inflammatory bowel disease by modulating gut microbiota and promoting MUC2 expression. Front Nutr. 2022;9:822020. 10.3389/fnut.2022.822020. 35252301 10.3389/fnut.2022.822020 PMC8892212

[104] Luo J, Li G, Hu W, Shen K, Huang X, Wang X, et al. The prognostic significance of visceral adiposity indices in conjunction with plasma trace elements in newly diagnosed Crohn’s disease. Int J Gen Med. 2025;18:5283–94. 40955325 10.2147/IJGM.S523034 PMC12433627

[105] Maukonen J, Kolho K-L, Paasela M, Honkanen J, Klemetti P, Vaarala O, et al. Altered Fecal microbiota in paediatric inflammatory bowel disease. J Crohns Colitis. 2015;9(12):1088–95. 26351391 10.1093/ecco-jcc/jjv147

[106] Lo Presti A, Zorzi F, Del Chierico F, Altomare A, Cocca S, Avola A, et al. Fecal and mucosal microbiota profiling in irritable bowel syndrome and inflammatory bowel disease. Front Microbiol. 2019;10:1655. 31379797 10.3389/fmicb.2019.01655 PMC6650632

[107] Pinto S, Šajbenová D, Benincà E, Nooij S, Terveer EM, Keller JJ, et al. Dynamics of gut microbiota after Fecal microbiota transplantation in ulcerative colitis: success linked to control of prevotellaceae. J Crohns Colitis. 2025;19(2):jjae137. 39225490 10.1093/ecco-jcc/jjae137 PMC11836888

[108] Bénard MV, de Goffau MC, Blonk J, Hugenholtz F, van Buuren J, Paramsothy S, et al. Gut microbiota features in relation to fecal microbiota transplantation outcome in ulcerative colitis: a Systematic review and meta-analysis. Clin Gastroenterol Hepatol. 2025;23(10):1719–36. 39442743 10.1016/j.cgh.2024.10.001

[109] Underwood W, Anthony R. AVMA guidelines for the Euthanasia of animals: 2020 edition. 2020th. Schaumburg: American Veterinary Medical Association; 2020.

