# Supplementary material for: Exploring new animal models of ulcerative colitis: evaluating chemical and patient-derived microbial triggers to advance translational relevance
Source: Lab Anim Res. 2026 Jun 8;42:21. doi: 10.1186/s42826-026-00283-9 (PMC13245015; doi:10.1186/s42826-026-00283-9)
Supplement: Supplementary file 1 — Supplementary Material 1 [file 42826_2026_283_MOESM1_ESM.pdf]

Supplementary Methods

1 Donor Screening and Selection

The donor screening process involved evaluating donors through anamnesis, clinical history, and laboratory testing. Donors were required to have a Body Mass Index (BMI) under 30, no history of gastrointestinal disorders other than UC (e.g., coeliac disease, irritable bowel syndrome, *Helicobacter pylori* infections), no antibiotic use in the previous three months, and no travel to third-world countries in the past six months. Laboratory testing included microbiological screening for *Salmonella*, *Shigella*, and *Clostridioides difficile* (GDH and toxins A, B), serological tests for HIV, hepatitis A–E, and *Treponema pallidum*, as well as bacterial pathogens such as *Escherichia coli* (various strains), *Vibrio cholerae*, and *Campylobacter spp*. Parasitological examinations targeted *Strongyloides stercoralis*, *Giardia intestinalis*, *Cryptosporidium spp.*, and *Entamoeba histolytica*, while virological screening assessed cytomegalovirus, rotavirus, norovirus I and II, and adenovirus 40 and 41. The chosen FMT donor was a 20-year-old female diagnosed with acute ulcerative colitis (Mayo score = 2). She was a non-smoker and abstinent. Her treatment history included mesalazine, with no prior use of antibiotics, corticosteroids, or biological therapies.

2 Fecal Microbiota Transplantation (FMT) Preparation

The stool sample was processed into FMT within 6 months of the screening tests and no more than 6 hours after collection. The protocol followed the method described by Lauko et al. [1]. Briefly, 50 g of stool was homogenized with 250 mL of saline using a mixer at 22,000 RPM in 4–5 intervals of 2 seconds each. The homogenate was then filtered through a series of sieves with pore sizes of 2.0, 1.0, 0.5, and 0.25 mm. The filtered mixture was centrifuged at 6000 RPM for 15 minutes at 4°C. Next, the supernatant was discarded, and the pellet was resuspended in 125 mL of saline. Glycerol was added as a cryoprotectant to a final concentration of 10% (v/v). The processed FMT was aliquoted and stored at -70°C until use in the experiment.

3 Animal Husbandry and Experimental Conditions

The study carried out on 96 specific pathogen-free (SPF) female BALB/c mice (6 weeks old) obtained from Velaz s.r.o. (Prague, Czech Republic). Upon arrival, the mice were housed in a gnotobiotic rearing facility, either a gnotobiotic rodent isolator EHRET THF 3271IE 101/97 (EHRET Labor-und Pharmatechnik GmbH Co.&KG, Emmendingen, Germany) or a two-sleeve breeding isolator (CBC, Ltd., Madison, WI, USA). Mice were kept in breeding polypropylene containers (365 mm × 207 mm × 140 mm), each accommodating 7–8 mice. All experimental animals were provided ad libitum access to irradiated complete feed formulated for ST-1 barrier-reared mice (Velaz Ltd., Prague, Czech Republic). Sterile, autoclaved water was provided in glass bottles, while irradiated bedding material (Lignocel 3-4 S, JRS, Rosenberg, Germany) was regularly replaced. Environmental conditions were maintained at 20–24 °C with relative humidity of 45–65%, ensured by HEPA-filtered air circulation (10–15 air exchanges per hour, 50–70 kPa overpressure, air flow 8–30 m^3). Noise levels did not exceed 45 dB, and light cycles mimicked natural circadian rhythms using a combination of internal neon fixtures and natural light. All glass and metal equipment was sterilized by autoclaving at 121 °C and 1.3 MPa for 30 minutes, while sanitary materials including cellulose wadding was gamma-irradiated (Bioster, Veverska Bityska, Czech Republic). All materials and equipment were additionally disinfected with 2% peracetic acid before placed into the breeding isolators.

4 Immunophenotyping of secondary lymphoid organs

Following euthanasia, the visceral cavity was opened, the spleen and bowels were removed and immediately placed in ice-cold Hanks' Balanced Salt Solution (HBSS) for preservation. The bowels were dissected and divided into three segments: small intestine, cecum, and large intestine.

4.1 Isolation of splenocytes

To isolate splenocytes, the spleen was perforated with a sterile needle and flushed with 5 mL of HBSS to release cells. The resulting cell suspension was collected in 15 mL Falcon tubes and centrifuged at 1,600 RPM for 5 minutes. The supernatant was discarded, and the pellet was resuspended in 1 mL of HBSS. This centrifugation and resuspension process was repeated three times in total. The final cell suspension was kept on ice until staining and analysis.

4.2 Isolation of cells from Peyer's patches

Peyer's patches were carefully excised from the small intestine and incubated with collagenase (100 IU/mL; Collagenase from *Clostridium histolyticum*) in RPMI 1640 medium (both from Sigma-Aldrich, St. Louis, MO, USA) in a water bath (37°C, 30 min, 220 RPM). Following incubation, the digested tissue was vortexed and filtered through nylon sieves (70 µm pore size; BD Falcon, Franklin Lakes, NJ, USA) with 5 mL of HBSS. Similarly to the isolation of splenocytes, the resulting cell suspension was centrifuged (1600 RPM, 4°C, 5 min), the supernatant discarded, and the pellet resuspended in 1 mL of HBSS (three times). The final cell suspension was stored on ice until staining.

4.3 Isolation of cells from colonic mucosa

Colon tissue intended for immunophenotyping was processed into a single-cell suspension following a modified version of the protocol by Bayne and Vonderheide (2013) [2]. A 2.5 cm section of colon was excised, minced into 1–2 mm² fragments in a sterile Petri dish, and transferred to wells of a 24-well plate (one or two wells depending on tissue volume). Each well was supplemented with 1 mL of collagenase solution containing Collagenase II (1.5 mg/mL), Collagenase IV (1 mg/mL), and Hyaluronidase IV (0.25 mg/mL) prepared in RPMI 1640 medium (Sigma-Aldrich, St. Louis, MO, USA). All enzymes were purchased from Worthington Biochemical Corporation (Lakewood, NJ, USA). Tissue digestion was carried out at 37°C for 45 minutes. Following incubation, the digested tissue was transferred to a 70 µm cell strainer (BD Falcon, Franklin Lakes, NJ, USA) positioned over a sterile Petri dish. The tissue was homogenized using the plunger of a 3-mL syringe, rinsed with 2–3 mL of medium, and passed through the strainer into the dish. The strained suspension was transferred to a 15-mL conical tube, centrifuged at 400g for 8 minutes at 4°C, and the supernatant discarded. The cell pellet was resuspended in approximately 8 mL of medium and filtered through a second 70 µm cell strainer into a fresh 50-mL conical tube. The filtrate was transferred to a 15-mL conical tube, centrifuged again under the same conditions, and resuspended in approximately 5 mL of medium.

5 Evaluation of microbial composition (16S sequencing)

Following an initial quality control assessment, amplicon libraries were constructed using universal primers 341F (5'-CCTAYGGGRBGCASCAG-3') and 806R (5'-GGACTACNNGGGTATCTAAT-3') targeting the V3-V4 regions of the 16S rRNA gene. These libraries were subsequently subjected to paired-end sequencing on the NovaSeq 6000 platform (Illumina, San Diego, CA, USA) in accordance with the manufacturer's guidelines. The sequencing run generated 2x250 bp reads, yielding approximately 30,000 raw tags per sample. Paired-end reads were merged using FLASH (V1.2.7) [3]. Quality filtering of the raw tags was performed to obtain high-quality clean tags according to the QIIME (V1.7.0) [4] quality control pipeline. Chimera sequences were identified and

removed by comparing the tags against the SILVA138 reference database [5] using the UCHIME algorithm [6], resulting in effective tags for downstream analysis. Effective tags were analyzed using the Uparse software (Uparse v7.0.1090) [7]. Sequences with ≥97% similarity were clustered into Operational Taxonomic Units (OTUs). For species annotation, representative sequences from each OTU were aligned to reference sequences from the SILVA138 Database using Mothur software [8]. The phylogenetic relationships among all OTU representative sequences were obtained using MUSCLE software (Version 3.8.31) [9]. Alpha diversity indices were calculated with QIIME. Principal Coordinates Analysis (PCoA) was visualized using the WGCNA [10], stats, and ggplot2 [11] packages in R software (Version 2.15.3) [12]. Unweighted Pair Group Method with Arithmetic Mean (UPGMA) clustering was performed using QIIME software. LEfSe analysis was carried out using the LEfSe software [13]. Permutational multivariate analysis of variance (Adonis) and Multi-Response Permutation Procedure (MRPP) were conducted in R using the vegan package (adonis and mrpp functions) [14]. Statistical comparisons (t-tests) and data visualizations were also performed in R.

6 Transcriptomic analysis of Colon mucosa (RNA-Seq)

Following an initial quality control assessment, mRNA was isolated from total RNA using poly-T oligo-attached magnetic beads to enrich for transcripts. First-strand cDNA synthesis was performed using random hexamer primers, followed by second-strand synthesis to generate double-stranded cDNA. The cDNA underwent adaptor ligation, digestion with Uracil-DNA Glycosylase, and PCR amplification to prepare a directional library. Sequencing was carried out on the NovaSeq 6000 platform (Illumina, San Diego, CA, USA), generating paired-end 150 bp reads in accordance with the manufacturer's protocols. Raw sequencing reads were quality-checked and aligned to the reference genome (*Mus musculus* GRCm38.p6, Ensembl accession GCA_000001635.8) using Hisat2 (v2.0.5) [15]. Mapped reads were assembled using StringTie (v1.3.3b) [16] in a reference-based approach, and gene-level read counts were obtained using featureCounts (v1.5.0-p3) [17]. Gene expression was quantified as FPKM (Fragments Per Kilobase of transcript sequence per Million base pairs sequenced). Differential expression analysis was performed with the DESeq2 R package (v1.20.0) [18], identifying differentially expressed genes with an adjusted p-value≤0.05 and a $\log_2$ FC (Fold Change) ≥1 (unless stated otherwise). Pathway enrichment analysis was conducted using the clusterProfiler R package (v 4.2.0) [19] with functional annotation based on the KEGG database[20–22]

1. Lauko S, Gancarcikova S, Hrckova G, et al (2023) Beneficial Effect of Faecal Microbiota Transplantation on Mild, Moderate and Severe Dextran Sodium Sulphate-Induced Ulcerative Colitis in a Pseudo Germ-Free Animal Model. Biomedicines 12:43
2. Bayne LJ, Vonderheide RH (2013) Multicolor Flow Cytometric Analysis of Immune Cell Subsets in Tumor-Bearing Mice. Cold Spring Harb Protoc 2013:pdb.prot077198
3. Magoč T, Salzberg SL (2011) FLASH: fast length adjustment of short reads to improve genome assemblies. Bioinformatics 27:2957–2963
4. Caporaso JG, Kuczynski J, Stombaugh J, et al (2010) QIIME allows analysis of high-throughput community sequencing data. Nat Methods 7:335–336
5. Quast C, Pruesse E, Yilmaz P, Gerken J, Schweer T, Yarza P, Peplies J, Glöckner FO (2012) The SILVA ribosomal RNA gene database project: improved data processing and web-based tools. Nucleic Acids Res 41:D590–D596
6. Edgar RC, Haas BJ, Clemente JC, Quince C, Knight R (2011) UCHIME improves sensitivity and speed of chimera detection. Bioinformatics 27:2194–2200
7. Edgar RC (2013) UPARSE: highly accurate OTU sequences from microbial amplicon reads. Nat Methods 10:996–998

8. Schloss PD, Westcott SL, Ryabin T, et al (2009) Introducing mothur: Open-Source, Platform-Independent, Community-Supported Software for Describing and Comparing Microbial Communities. Appl Environ Microbiol 75:7537–7541
9. Edgar RC (2004) MUSCLE: multiple sequence alignment with high accuracy and high throughput. Nucleic Acids Res 32:1792–1797
10. Langfelder P, Horvath S (2008) WGCNA: an R package for weighted correlation network analysis. BMC Bioinformatics 9:559
11. Wickham H (2016) ggplot2. https://doi.org/10.1007/978-3-319-24277-4
12. R Core Team (2023) R: A Language and Environment for Statistical Computing.
13. Segata N, Izard J, Waldron L, Gevers D, Miropolsky L, Garrett WS, Huttenhower C (2011) Metagenomic biomarker discovery and explanation. Genome Biol 12:R60
14. Oksanen J, Simpson GL, Blanchet FG, et al (2001) vegan: Community Ecology Package. CRAN: Contributed Packages. https://doi.org/10.32614/CRAN.package.vegan
15. Kim D, Langmead B, Salzberg SL (2015) HISAT: a fast spliced aligner with low memory requirements. Nat Methods 12:357–360
16. Pertea M, Pertea GM, Antonescu CM, Chang T-C, Mendell JT, Salzberg SL (2015) StringTie enables improved reconstruction of a transcriptome from RNA-seq reads. Nat Biotechnol 33:290–295
17. Liao Y, Smyth GK, Shi W (2014) featureCounts: an efficient general purpose program for assigning sequence reads to genomic features. Bioinformatics 30:923–930
18. Love MI, Huber W, Anders S (2014) Moderated estimation of fold change and dispersion for RNA-seq data with DESeq2. Genome Biol 15:550
19. Yu G, Wang L-G, Han Y, He Q-Y (2012) clusterProfiler: an R Package for Comparing Biological Themes Among Gene Clusters. OMICS 16:284–287
20. Kanehisa M (2000) KEGG: Kyoto Encyclopedia of Genes and Genomes. Nucleic Acids Res 28:27–30
21. Kanehisa M (2019) Toward understanding the origin and evolution of cellular organisms. Protein Science 28:1947–1951
22. Kanehisa M, Furumichi M, Sato Y, Matsuura Y, Ishiguro-Watanabe M (2025) KEGG: biological systems database as a model of the real world. Nucleic Acids Res 53:D672–D677
