# Supplementary material for: Exploring new animal models of ulcerative colitis: evaluating chemical and patient-derived microbial triggers to advance translational relevance
Source: Lab Anim Res. 2026 Jun 8;42:21. doi: 10.1186/s42826-026-00283-9 (PMC13245015; doi:10.1186/s42826-026-00283-9)
Supplement: Supplementary file 2 — Supplementary Material 2 [file 42826_2026_283_MOESM2_ESM.pdf]

Additional Table 1: Antibody mix1: Spleen and Peyer's Patches

Antibody	Clone	Isotype	Fluorophore
anti-B220	RA3-6B2	IgG2a, κ	APC
anti-CD3	17A2	IgG2b, κ	PerCP-eFluor710
anti-CD4	GK 1.5	IgG2b, κ	FITC
anti-CD45	30-F11	IgG2b, κ	APC-Cy7
anti-CD49b	DX5	IgM, κ	APC
anti-CD8a	53-6.7	IgG2a, κ	R-PE
