# Supplementary material for: Exploring new animal models of ulcerative colitis: evaluating chemical and patient-derived microbial triggers to advance translational relevance
Source: Lab Anim Res. 2026 Jun 8;42:21. doi: 10.1186/s42826-026-00283-9 (PMC13245015; doi:10.1186/s42826-026-00283-9)
Supplement: Supplementary file 3 — Supplementary Material 3 [file 42826_2026_283_MOESM3_ESM.pdf]

Additional Table 2: Antibody mix2: Colon mucosa

Antibody	Clone	Isotype	Fluorophore
Anti-CD11b	M1/70	IgG2b, κ	PerCP-Cy5.5
Anti-CD19	1D3	IgG2a, κ	APC
Anti-CD3e	145-2C11	IgG	PerCP-Cy5.5
Anti-CD4	GK 1.5	IgG2b, κ	APC-eFluor® 780
Anti-CD45	30-F11	IgG2b, κ	FITC
Anti-CD45	30-F11	IgG2b, κ	PE-Cy7
Anti-CD49b	DX5	IgM, κ	PE
Anti-CD8a	53-6.7	IgG2a, κ	PE-Cy7
Anti-F4/80	BM8	IgG2a, κ	FITC
Anti-Ly-6G	RB6-8C5	IgG2b, κ	APC- eFluor® 780
