# Supplementary material for: Exploring new animal models of ulcerative colitis: evaluating chemical and patient-derived microbial triggers to advance translational relevance
Source: Lab Anim Res. 2026 Jun 8;42:21. doi: 10.1186/s42826-026-00283-9 (PMC13245015; doi:10.1186/s42826-026-00283-9)
Supplement: Supplementary file 4 — Supplementary Material 4 [file 42826_2026_283_MOESM4_ESM.pdf]

Additional Table 3: Selected genes for differential gene expression analysis

**Gene symbol**	**Gene name**
**Tight junctions**	
F11r	F11 receptor / Junctional adhesion molecule 1
Jam2	Junction adhesion molecule 2
Jam3	Junction adhesion molecule 3
Igsf5	Immunoglobin superfamily member 5 / Junctional adhesion molecule 4
Crb3	Crumbs family member 3
Cxadr	Coxsackie virus and adenovirus receptor
Bves	Blood vessel epicardial substance / Popeye domain cAMP effector 1
Cldn1	Claudin 1
Cldn2	Claudin 2
Cldn3	Claudin 3
Cldn4	Claudin 4
Cldn5	Claudin 5
Cldn6	Claudin 6
Cldn7	Claudin 7
Cldn8	Claudin 8
Cldn9	Claudin 9
Cldn10	Claudin 10
Cldn11	Claudin 11
Cldn12	Claudin 12
Cldn13	Claudin 13
Cldn14	Claudin 14
Cldn15	Claudin 15
Cldn16	Claudin 16
Cldn17	Claudin 17
Cldn18	Claudin 18
Cldn19	Claudin 19
Cldn20	Claudin 20
Cldn22	Claudin 22
Cldn23	Claudin 23
Cldn24	Claudin 24
Cldn25	Claudin 25 / Claudin 21
Tmem114	Transmembrane protein 114 / Claudin 26
Tmem235	Transmembrane protein 235 / Claudin 27
Ocln	Occludin
Marveld2	MARVEL domain containing 2 / Tricellulin
Marveld3	MARVEL domain containing 3
Tjp1	Tight junction protein 1 / Zonula Occludens Protein 1
Tjp2	Tight junction protein 2 / Zonula Occludens Protein 2
Tjp3	Tight junction protein 3 / Zonula Occludens Protein 3
Afdn	Afadin / Adherens junction formation factor
Ctnna1	Catenin alpha 1
Pard3	Par-3 family cell polarity regulator / Partitioning defective 3 homolog

Pals1	Protein associated with LIN7 1
Patj	Crumbs cell polarity complex component / Pals1-associated tight junction protein
Cgn	Cingulin
**Mucins**	
Muc1	Mucin 1
Muc2	Mucin 2
Muc4	Mucin 4
Muc5b	Mucin 5b
Muc12	Mucin 12
Muc13	Mucin 13
Muc15	Mucin 15
Muc16	Mucin 16
Muc17	Mucin 17 / Mucin 3
Muc20	Mucin 20
Muc21	Mucin 21
**Chemokines**	
Ccl2	C-C motif chemokine ligand 2
Ccl3	C-C motif chemokine ligand 3
Ccl4	C-C motif chemokine ligand 4
Ccl5	C-C motif chemokine ligand 5
Ccl7	C-C motif chemokine ligand 7
Ccl8	C-C motif chemokine ligand 8
Ccl11	C-C motif chemokine ligand 11
Ccl17	C-C motif chemokine ligand 17
Ccl19	C-C motif chemokine ligand 19
Ccl20	C-C motif chemokine ligand 20
Ccl22	C-C motif chemokine ligand 22
Ccl25	C-C motif chemokine ligand 25
Ccl28	C-C motif chemokine ligand 28
Cxcl1	C-X-C motif chemokine ligand 1
Cxcl2	C-X-C motif chemokine ligand 2
Cxcl3	C-X-C motif chemokine ligand 3
Cxcl5	C-X-C motif chemokine ligand 5
Cxcl9	C-X-C motif chemokine ligand 9
Cxcl10	C-X-C motif chemokine ligand 10
Cxcl11	C-X-C motif chemokine ligand 11
Cxcl15	C-X-C motif chemokine ligand 15
Cx3cl1	
**Cytokines**	
Tnf	Tumor necrosis factor
Il1a	Interleukin 1 alpha
Il1b	Interleukin 1 beta
Il2	Interleukin 2
Il3	Interleukin 3
Il4	Interleukin 4

Il5	Interleukin 5
Il6	Interleukin 6
Il7	Interleukin 7
Il9	Interleukin 9
Il10	Interleukin 10
Il11	Interleukin 11
Il12a	Interleukin 12a
Il12b	Interleukin 12b
Il13	Interleukin 13
Il17a	Interleukin 17a
Il17f	Interleukin 17f
Il18	Interleukin 18
Il20	Interleukin 20
Il21	Interleukin 21
Il22	Interleukin 22
Il23a	Interleukin 23a
Il27	Interleukin 27
Il33	Interleukin 33
Tgfb1	Transforming growth factor beta 1
Tgfb2	Transforming growth factor beta 2
Tgfb3	Transforming growth factor beta 3
Ifna	Interferon alpha
Ifnb1	Interferon beta 1
Ifnl2	Interferon beta 2
Ifng	Interferon gamma
Lif	Leukemia inhibitory factor
Osm	Oncostatin M
Tnfsf15	Tumor necrosis factor superfamily member 15 / TNF superfamily ligand TL1A
Csf2	Colony stimulating factor 2
