# Supplementary material for: Exploring new animal models of ulcerative colitis: evaluating chemical and patient-derived microbial triggers to advance translational relevance
Source: Lab Anim Res. 2026 Jun 8;42:21. doi: 10.1186/s42826-026-00283-9 (PMC13245015; doi:10.1186/s42826-026-00283-9)
Supplement: Supplementary file 6 — Supplementary Material 6 [file 42826_2026_283_MOESM6_ESM.pdf]

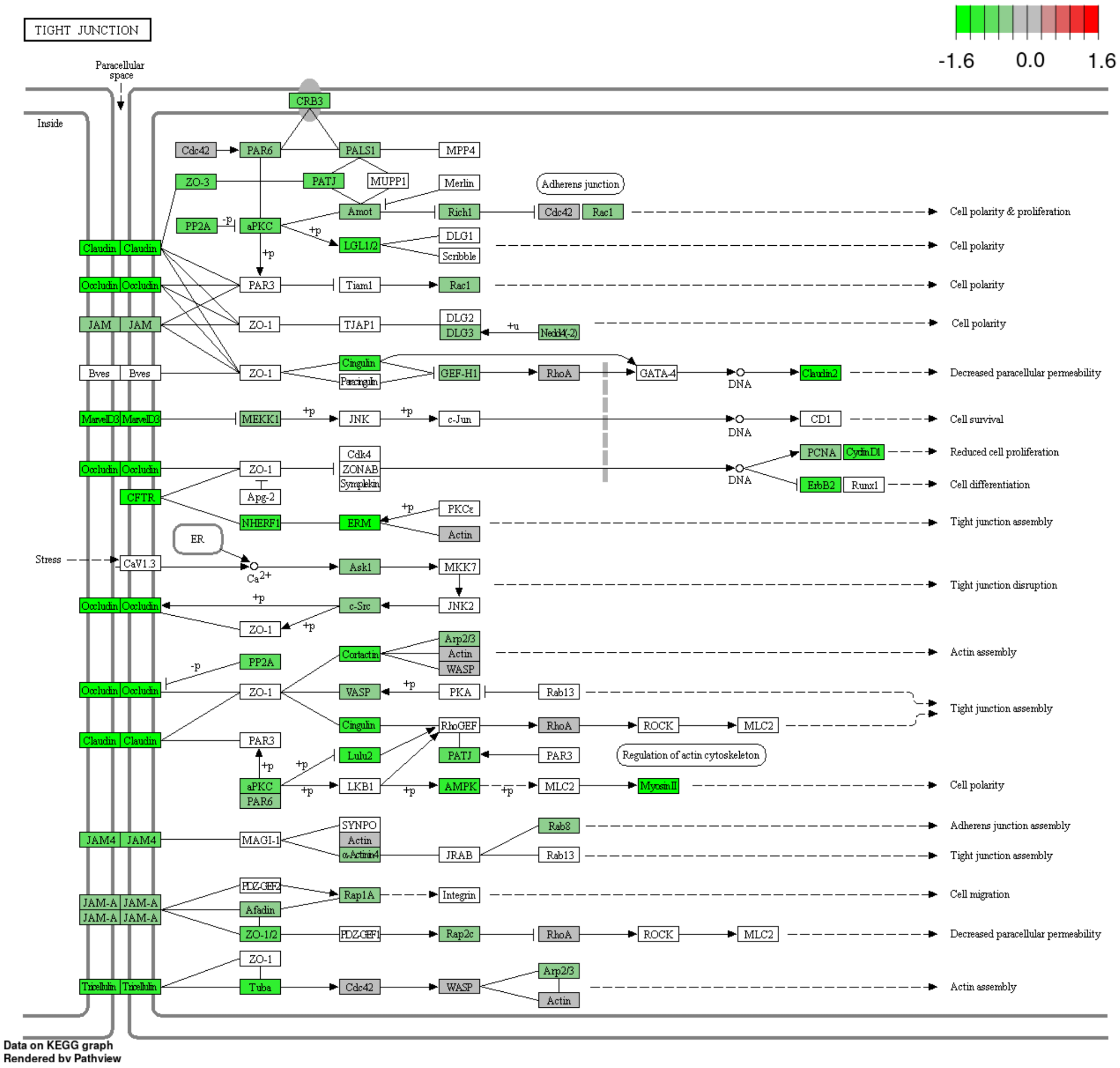

Additional Figure 2: KEGG Tight Junction Pathway (mmu04530) with differentially expressed genes between COMB and C0 groups highlighted. The color gradient scale in the top-right corner represents Log2FC values. KEGG pathway map was adapted from KEGG Kanehisa Laboratories [1–3], with permission under CC BY 4.0 license.

1. Kanehisa M (2000) KEGG: Kyoto Encyclopedia of Genes and Genomes. Nucleic Acids Res 28:27–30
2. Kanehisa M (2019) Toward understanding the origin and evolution of cellular organisms. Protein Science 28:1947–1951
3. Kanehisa M, Furumichi M, Sato Y, Matsuura Y, Ishiguro-Watanabe M (2025) KEGG: biological systems database as a model of the real world. Nucleic Acids Res 53:D672–D677
