# Supplementary material for: Exploring new animal models of ulcerative colitis: evaluating chemical and patient-derived microbial triggers to advance translational relevance
Source: Lab Anim Res. 2026 Jun 8;42:21. doi: 10.1186/s42826-026-00283-9 (PMC13245015; doi:10.1186/s42826-026-00283-9)
Supplement: Supplementary file 7 — Supplementary Material 7 [file 42826_2026_283_MOESM7_ESM.docx]

**Additional file legends**

**Additional file 1:**

File name and format: Additional_file1.pdf

Title of data: Supplementary methods

Description of data: Supplementary information regarding the detailed methods of donor screening and selection, FMT preparation, animal husbandry and experimental conditions, immunophenotyping of secondary lymphoid organs (preparation of single cell suspensions), evaluation of microbial composition (16S sequencing) and transcriptomic analysis of colon mucosa (RNA-Seq)

**Additional file 2:**

File name and format: Additional_file2.pdf

Title of data: Additional Table 1: Antibody mix1: Spleen and Peyer’s Patches

Description of data: Table regarding the composition of the antibody mix used to stain single cell suspensions from Peyer’s Patches and spleen

**Additional file 3:**

File name and format: Additional_file3.pdf

Title of data: Additional Table 2: Antibody mix2: Colon mucosa

Description of data: Table regarding the composition of the antibody mix used to stain single cell suspensions from colon mucosa

**Additional file 4:**

File name and format: Additional_file4.pdf

Title of data: Additional Table 3: Selected genes for differential gene expression analysis

Description of data: Panel of 114 genes curated from literature intended for targeted differential gene expression analysis

**Additional file 5:**

File name and format: Additional_file5.pdf

Title of data: Additional Figure 1

Description of data: KEGG Tight Junction Pathway (mmu04530) with differentially expressed

genes between FMT and C0 groups highlighted. The colour gradient scale in the top-right corner

represents Log2FC values. KEGG pathway map was adapted from KEGG Kanehisa Laboratories

with permission under CC BY 4.0 license.

**Additional file 6:**

File name and format: Additional_file6.pdf

Title of data: Additional Figure 2

Description of data: KEGG Tight Junction Pathway (mmu04530) with differentially expressed

genes between COMB and C0 groups highlighted. The colour gradient scale in the top-right corner

represents Log2FC values. KEGG pathway map was adapted from KEGG Kanehisa Laboratories

with permission under CC BY 4.0 license.
